# Supplementary figures and images for: Identification of a third myosin-5a-melanophilin interaction that mediates the association of myosin-5a with melanosomes
Source: eLife. 2024 Jun 20;13:RP93662. doi: 10.7554/eLife.93662 (PMC11189624; doi:10.7554/eLife.93662)

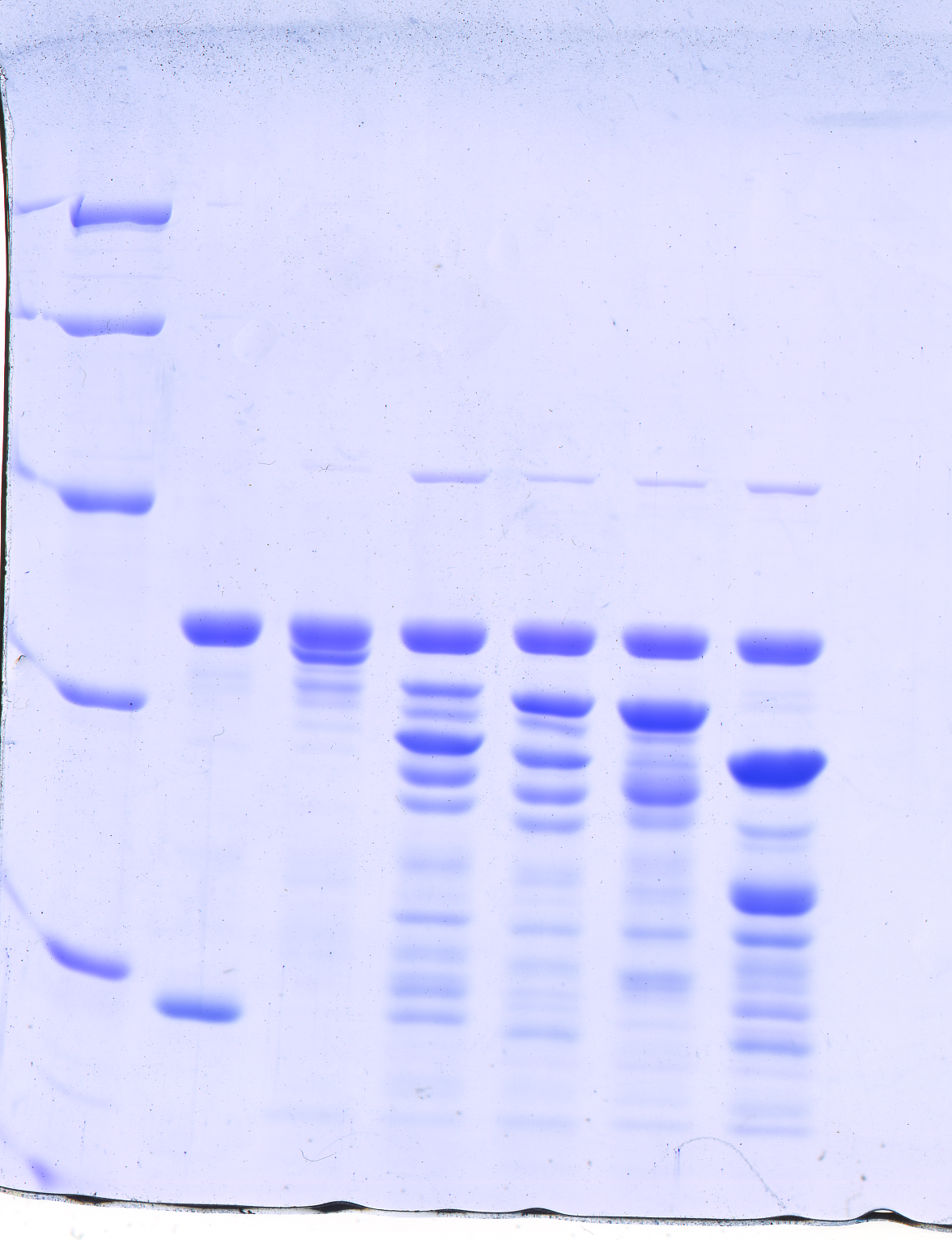

Supplement: Figure 1—source data 1. [file elife-93662-fig1-data1.zip › Figure 1-Source data 1/Original files for Figure 1C/Mlph-ABD N truncation pulldown Myo5a-MTD Input.tif]

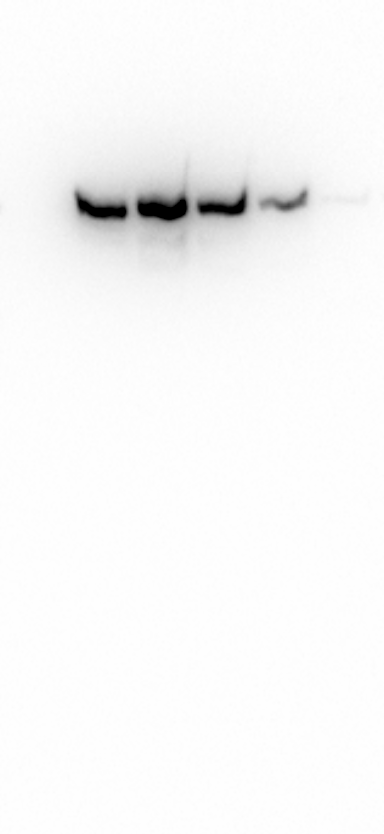

Supplement: Figure 1—source data 1. [file elife-93662-fig1-data1.zip › Figure 1-Source data 1/Original files for Figure 1C/Mlph-ABD N truncation pulldown Myo5a-MTD WB.tif]

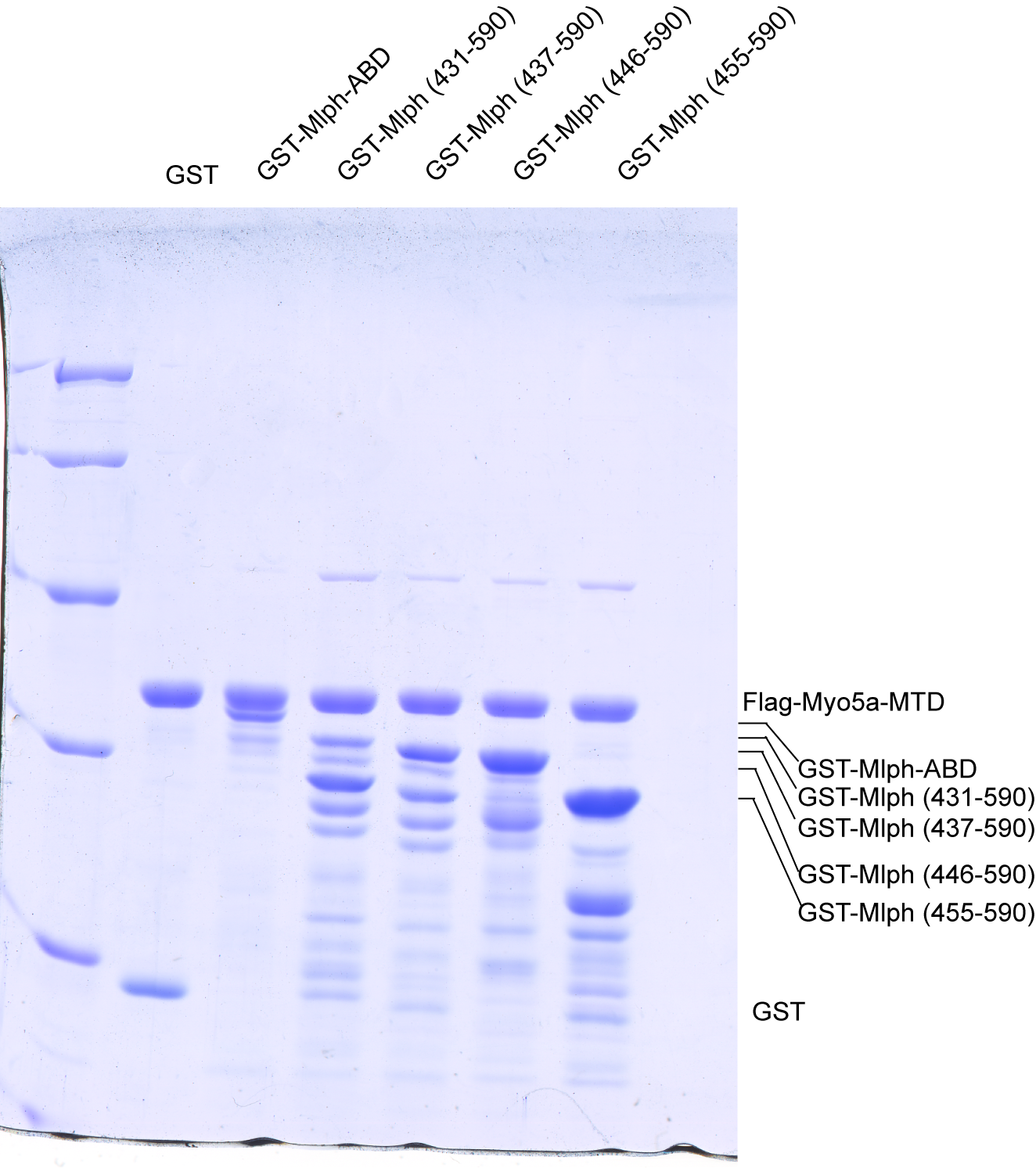

Supplement: Figure 1—source data 1. [file elife-93662-fig1-data1.zip › Figure 1-Source data 1/Uncropped gels and blots for Figure 1C/Mlph-ABD N truncation pulldown Myo5a-MTD Input.tif]

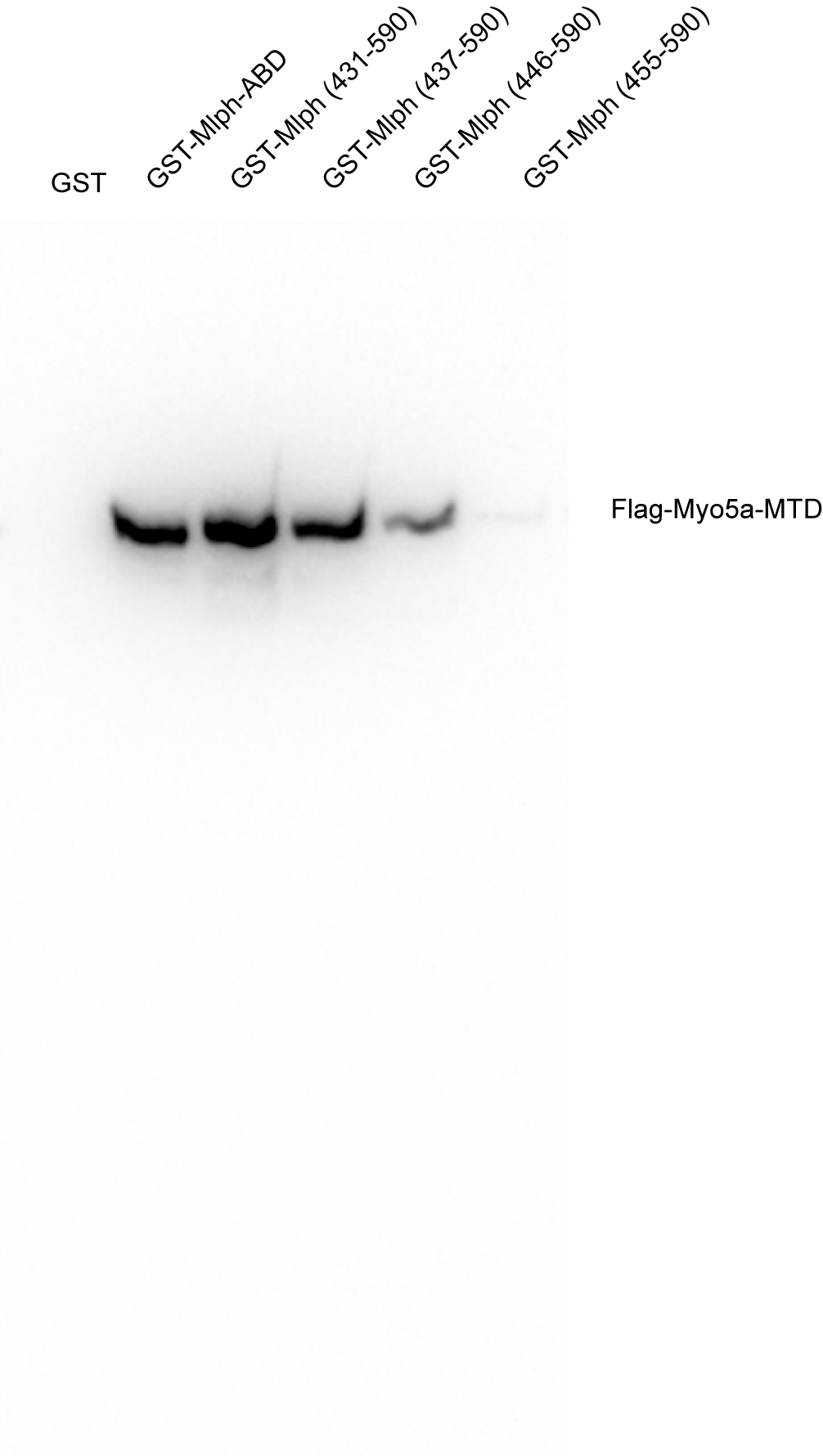

Supplement: Figure 1—source data 1. [file elife-93662-fig1-data1.zip › Figure 1-Source data 1/Uncropped gels and blots for Figure 1C/Mlph-ABD N truncation pulldown Myo5a-MTD WB.tif]

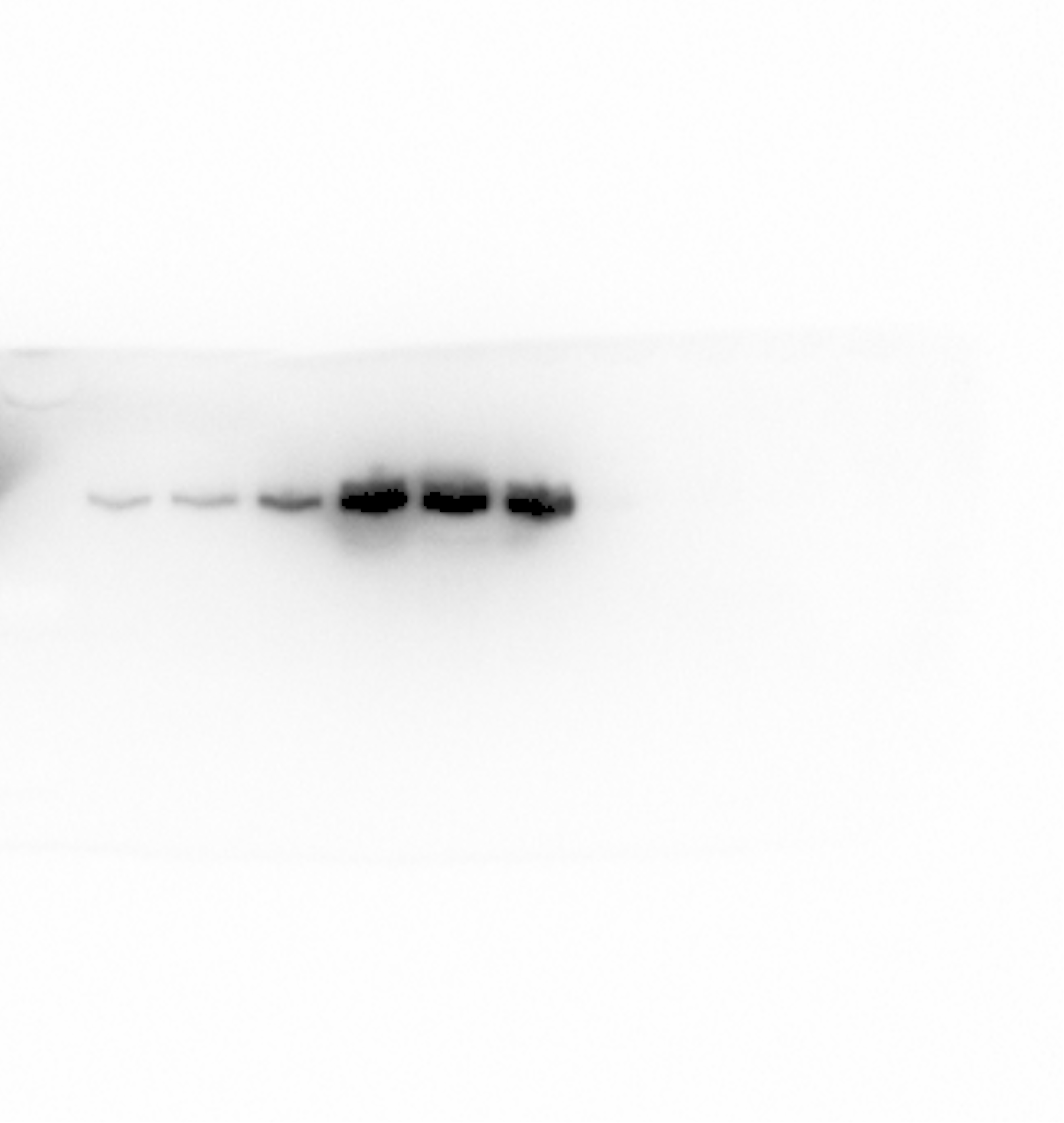

Supplement: Figure 1—source data 2. [file elife-93662-fig1-data2.zip › Figure 1-Source data 2/Original files for Figure 1D/Mlph-ABD C truncation pulldown Myo5a-MTD westerm blot.tif]

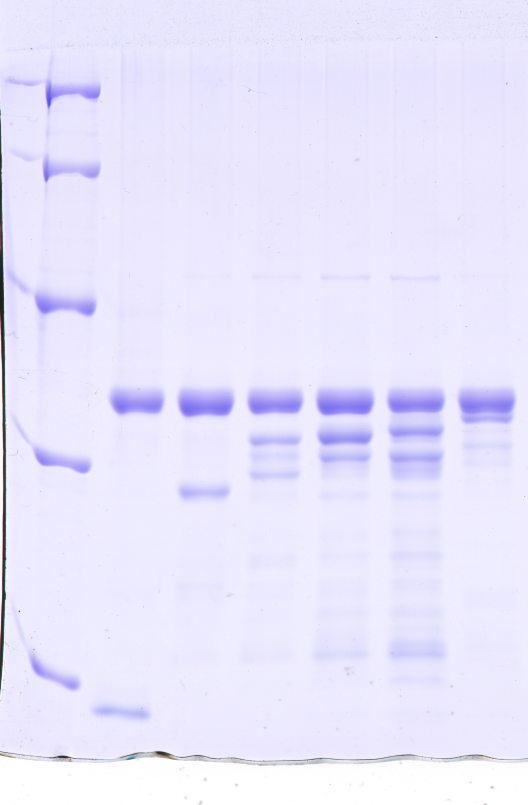

Supplement: Figure 1—source data 2. [file elife-93662-fig1-data2.zip › Figure 1-Source data 2/Original files for Figure 1D/Mlph-ABD C truncation pulldown Myo5a-MTD Input.tif]

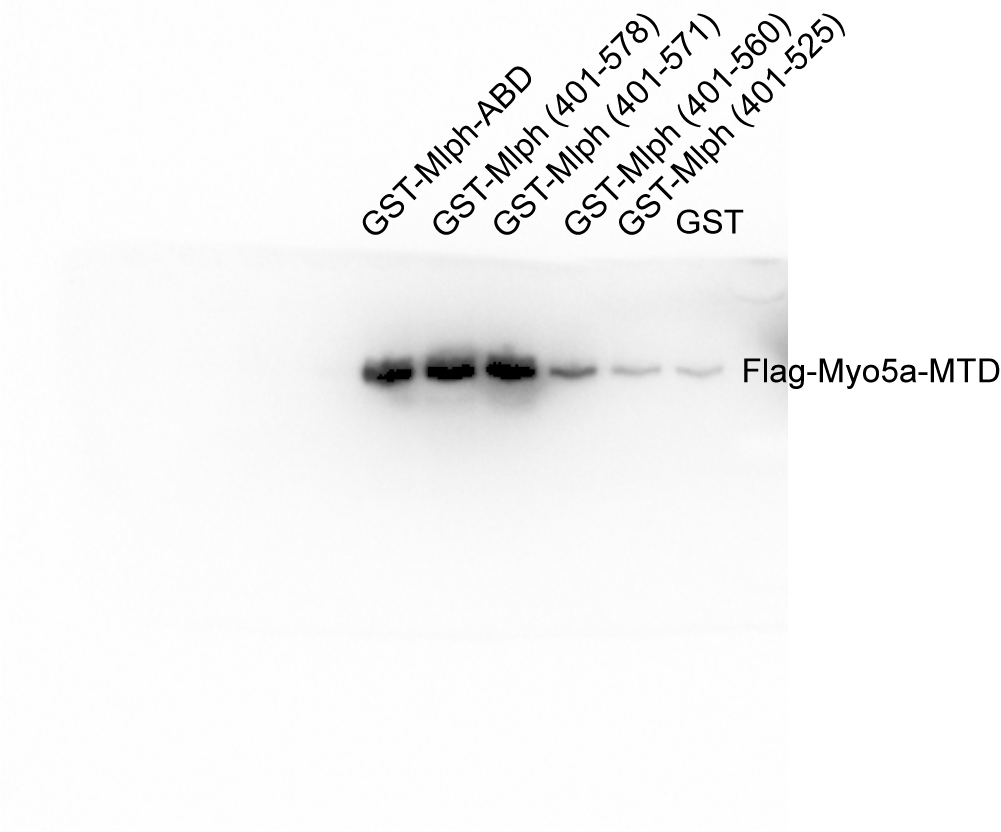

Supplement: Figure 1—source data 2. [file elife-93662-fig1-data2.zip › Figure 1-Source data 2/Uncropped gels and blots for Figure 1D/Mlph-ABD C truncation pulldown Myo5a-MTD westerm blot.tif]

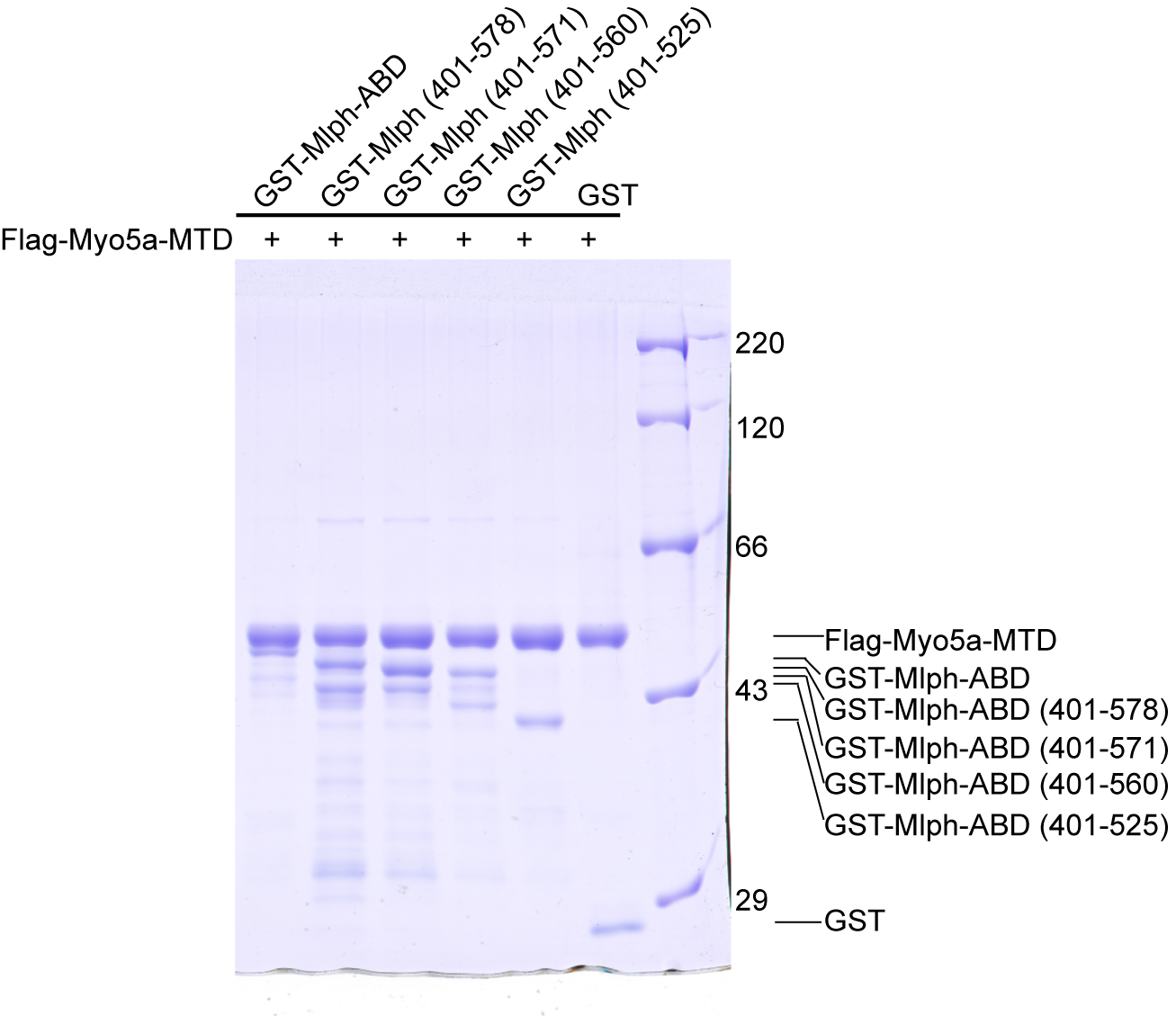

Supplement: Figure 1—source data 2. [file elife-93662-fig1-data2.zip › Figure 1-Source data 2/Uncropped gels and blots for Figure 1D/Mlph-ABD C truncation pulldown Myo5a-MTD Input.tif]

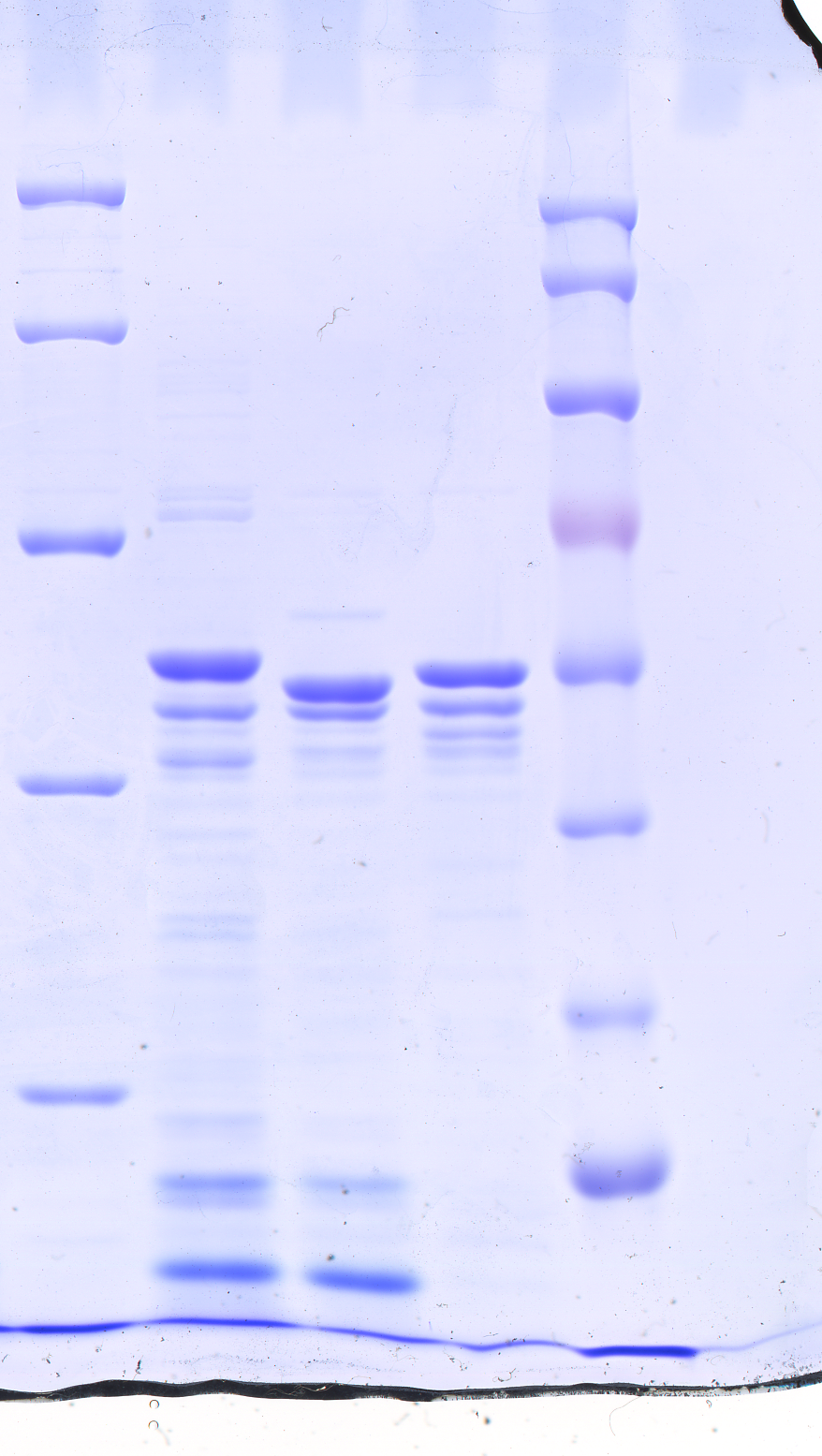

Supplement: Figure 2—source data 1. [file elife-93662-fig2-data1.zip › Figure 2-Source data 1/Original files for Figure 2B/Mlph-ABD pulldown M5a-MTD ΔF ΔG Input.tif]

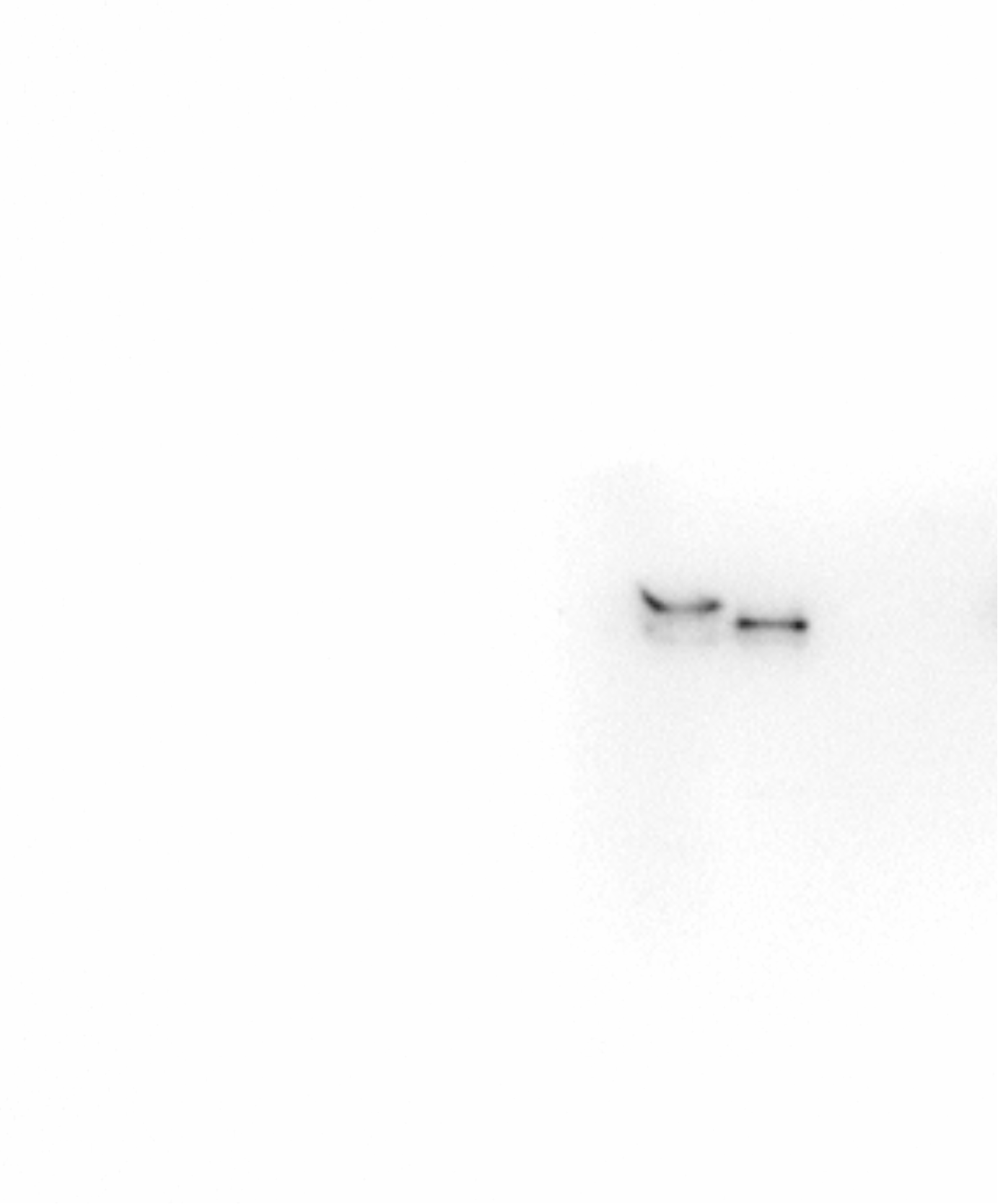

Supplement: Figure 2—source data 1. [file elife-93662-fig2-data1.zip › Figure 2-Source data 1/Original files for Figure 2B/Mlph-ABD pulldown M5a-MTD ΔF ΔG WB.tif]

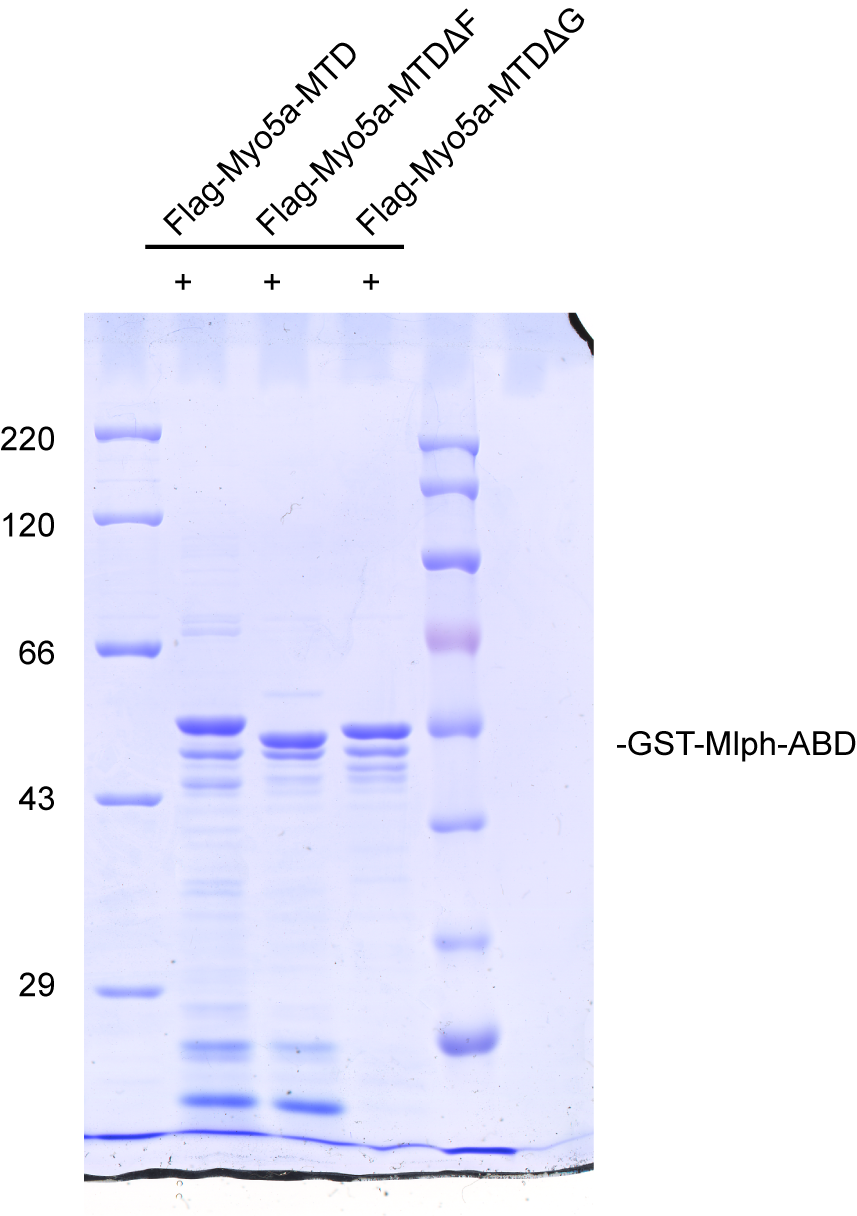

Supplement: Figure 2—source data 1. [file elife-93662-fig2-data1.zip › Figure 2-Source data 1/Uncropped gels and blots for Figure 2B/Mlph-ABD pulldown M5a-MTD ΔF ΔG Input.tif]

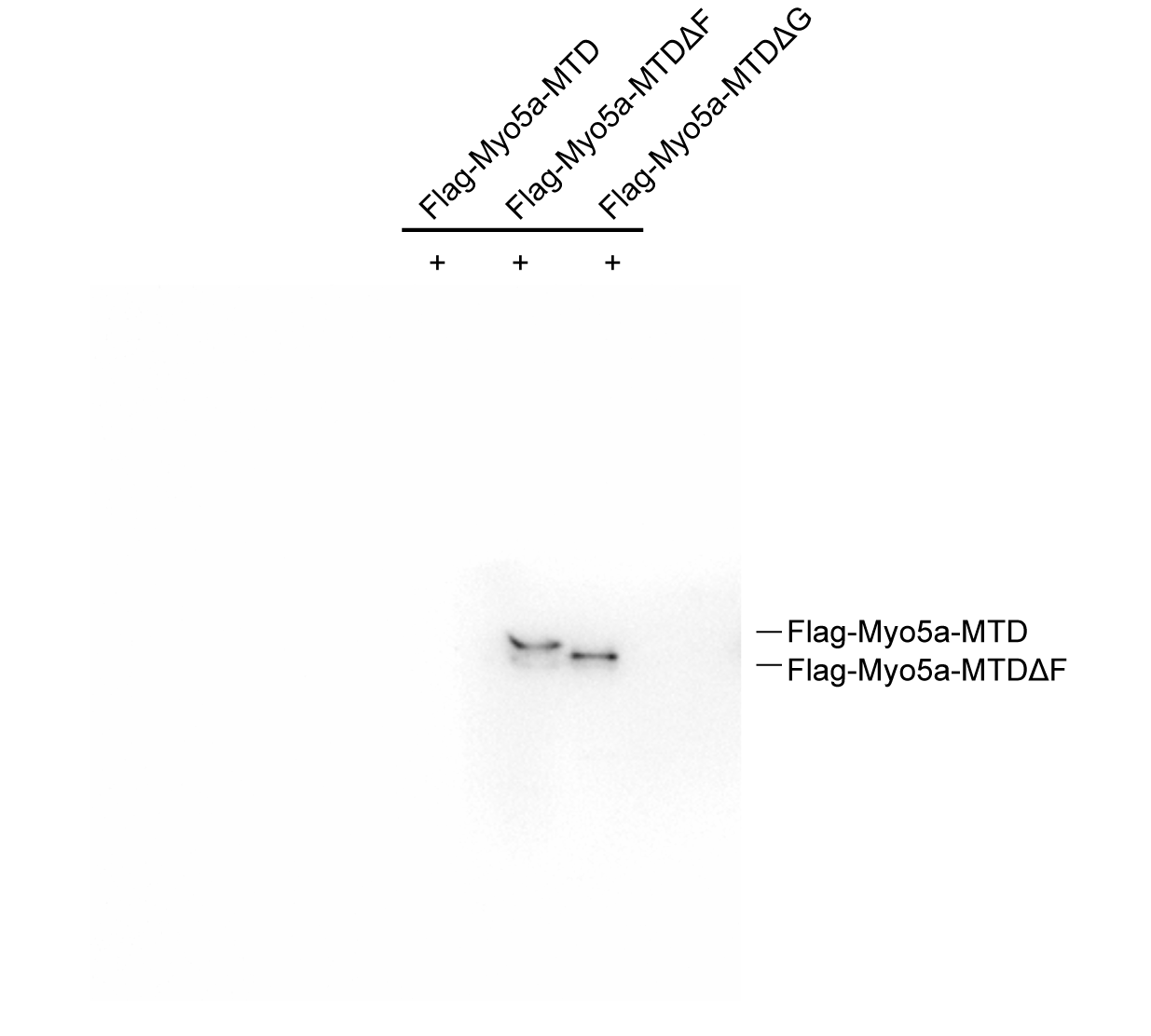

Supplement: Figure 2—source data 1. [file elife-93662-fig2-data1.zip › Figure 2-Source data 1/Uncropped gels and blots for Figure 2B/Mlph-ABD pulldown M5a-MTD ΔF ΔG WB.tif]

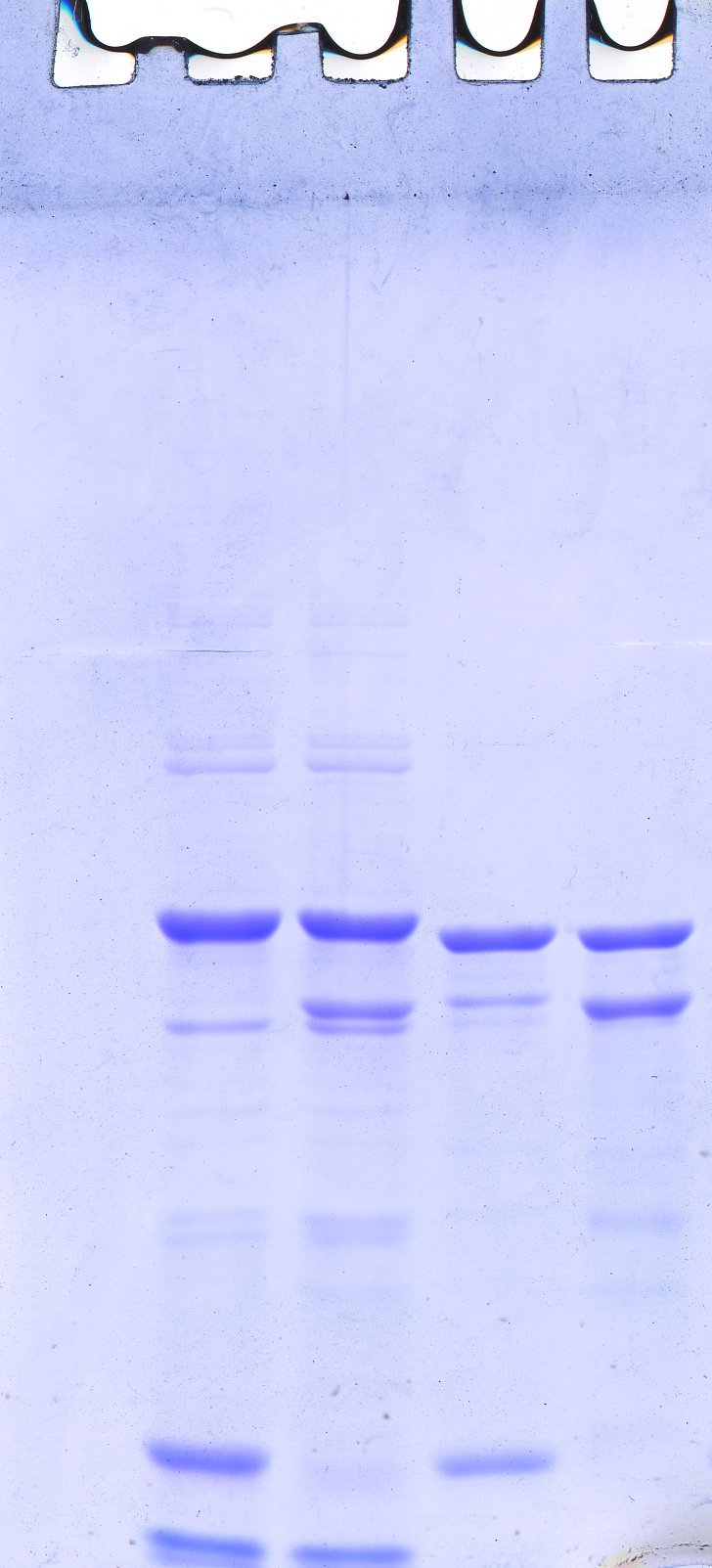

Supplement: Figure 2—source data 2. [file elife-93662-fig2-data2.zip › Figure 2-Source data 2/Original files for Figure 2C/Mlph-EFBD pulldown M5a-MTD and MTD ΔG Input.tif]

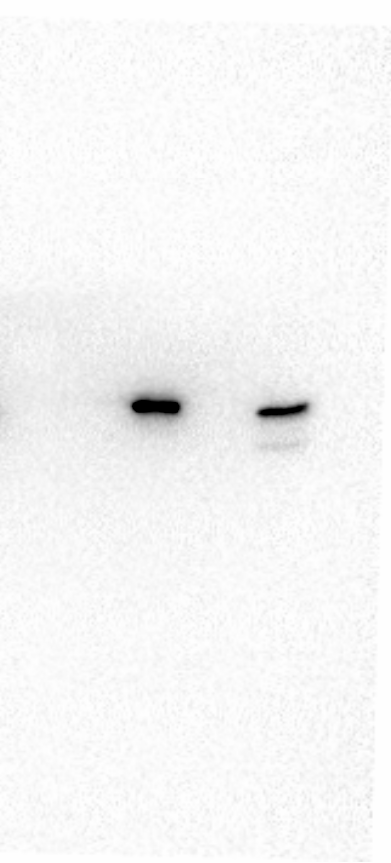

Supplement: Figure 2—source data 2. [file elife-93662-fig2-data2.zip › Figure 2-Source data 2/Original files for Figure 2C/Mlph-EFBD pulldown M5a-MTD and MTD ΔG WB.tif]

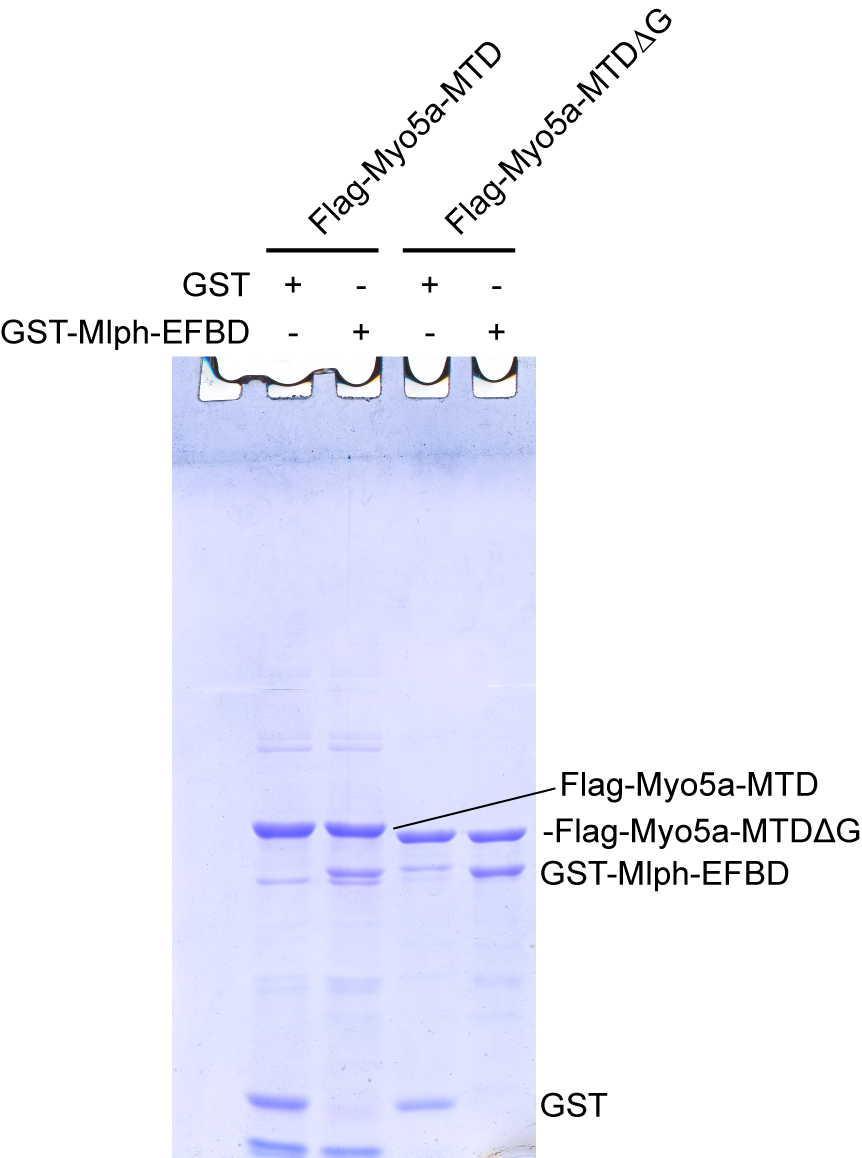

Supplement: Figure 2—source data 2. [file elife-93662-fig2-data2.zip › Figure 2-Source data 2/Uncropped gels and blots for Figure 2C/Mlph-EFBD pulldown M5a-MTD and MTD ΔG Input.tif]

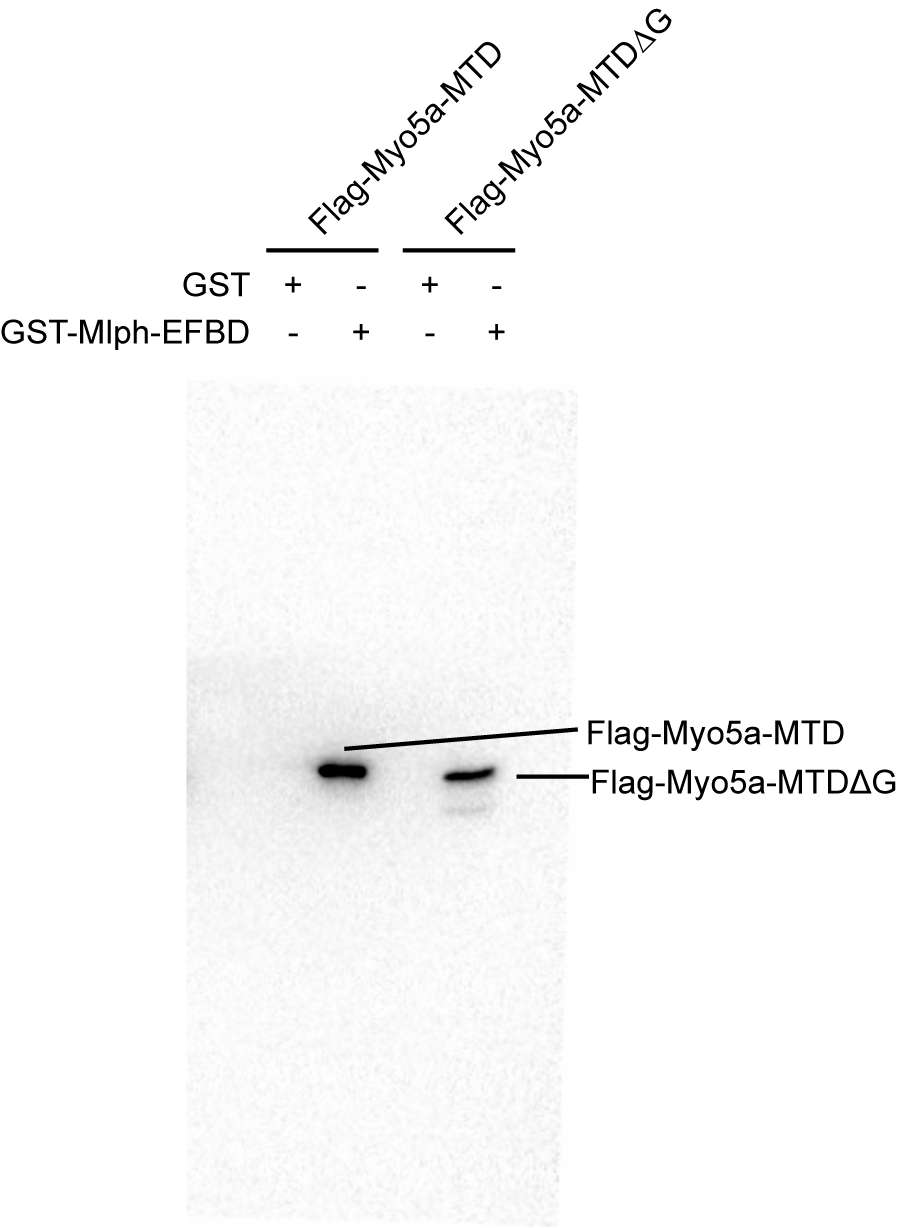

Supplement: Figure 2—source data 2. [file elife-93662-fig2-data2.zip › Figure 2-Source data 2/Uncropped gels and blots for Figure 2C/Mlph-EFBD pulldown M5a-MTD and MTD ΔG WB.tif]

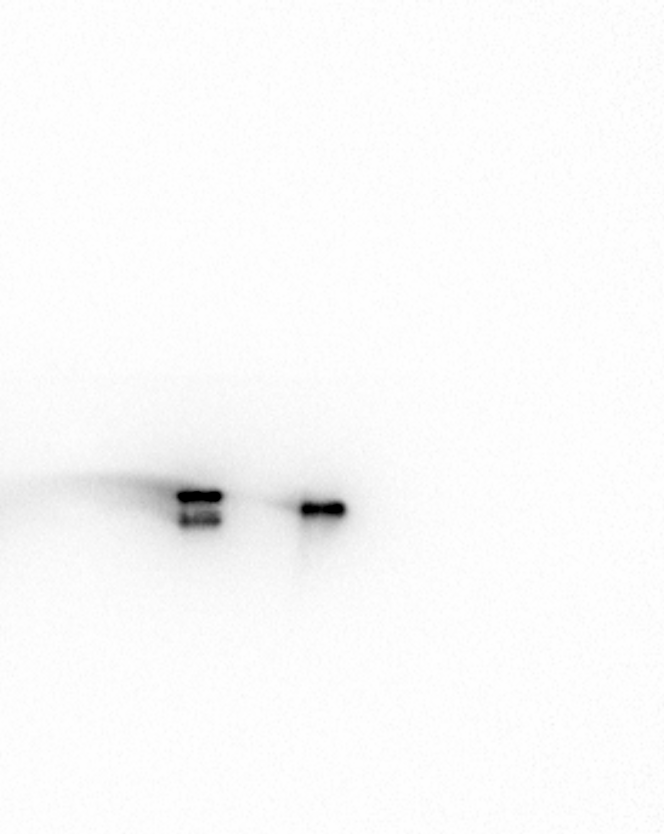

Supplement: Figure 2—figure supplement 1—source data 1. [file elife-93662-fig2-figsupp1-data1.zip › Figure 2-figure supplement Source data 1/Original files for Figure 2 supplement A/Mlph-ABD pulldown Myo5a-MTD ecoli and sf9 wb.tif]

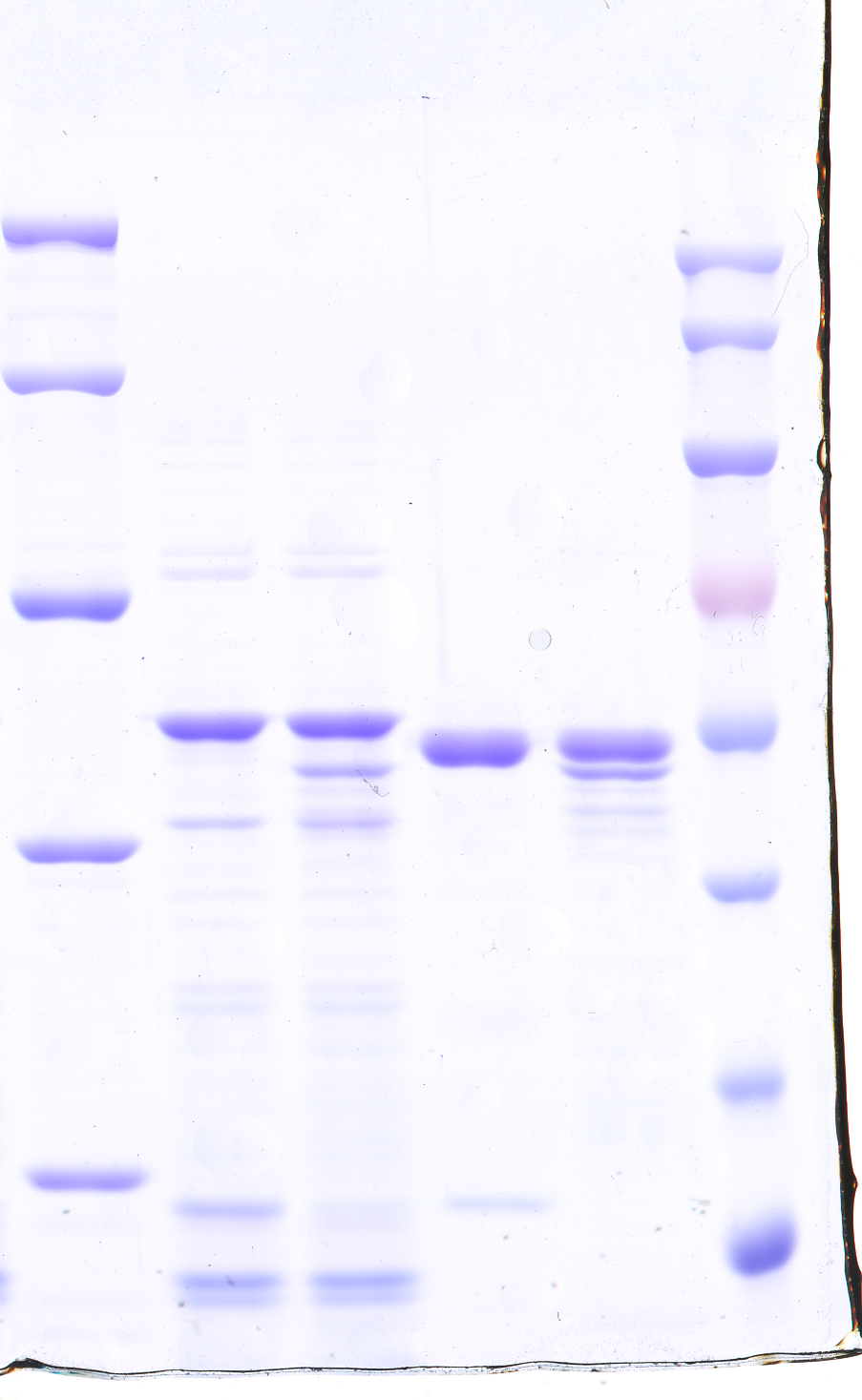

Supplement: Figure 2—figure supplement 1—source data 1. [file elife-93662-fig2-figsupp1-data1.zip › Figure 2-figure supplement Source data 1/Original files for Figure 2 supplement A/Mlph-ABD pulldown Myo5a-MTD ecoli and sf9.tif]

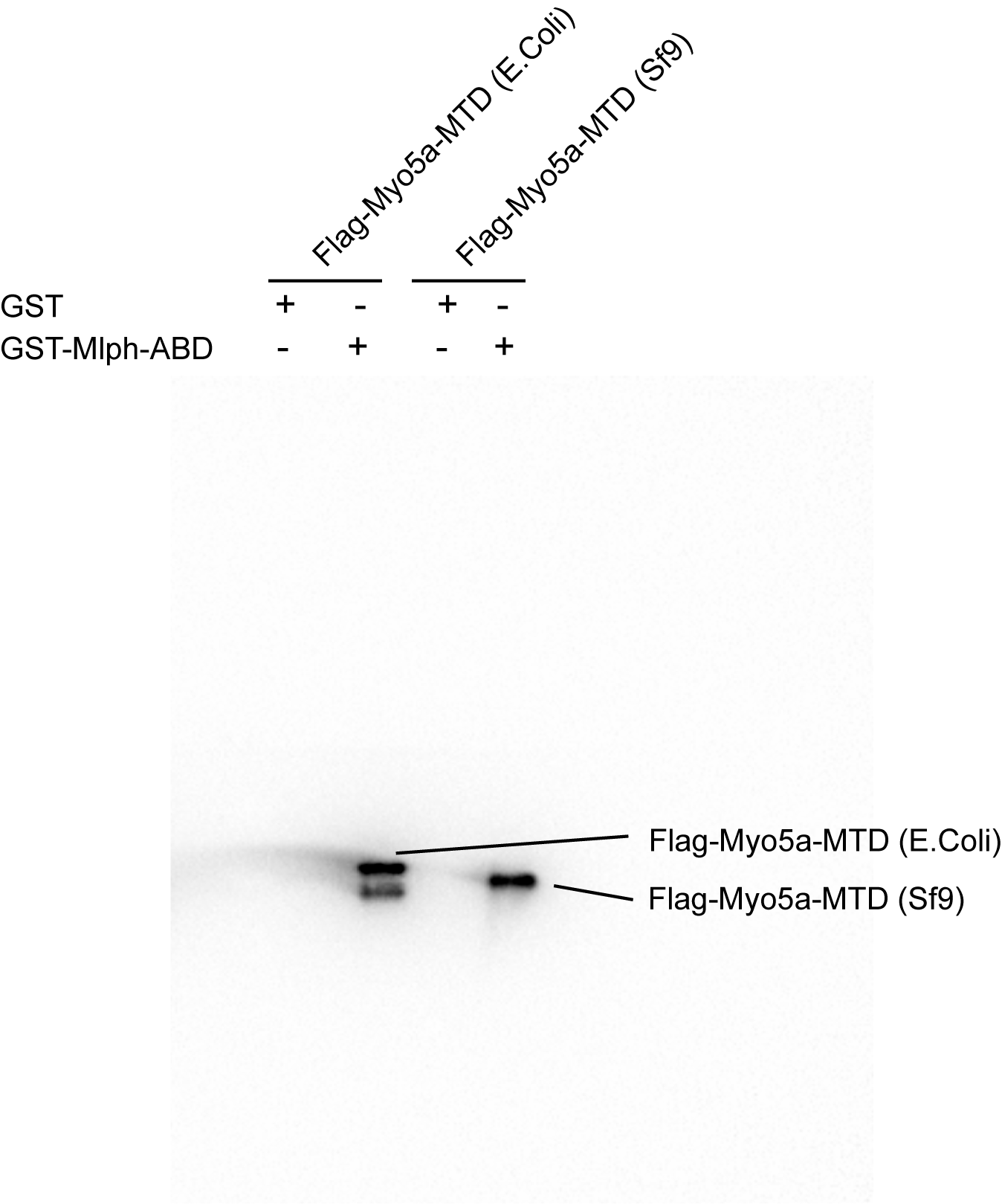

Supplement: Figure 2—figure supplement 1—source data 1. [file elife-93662-fig2-figsupp1-data1.zip › Figure 2-figure supplement Source data 1/Uncropped gels and blots for Figure 2 supplement A/Mlph-ABD pulldown Myo5a-MTD ecoli and sf9 wb.tif]

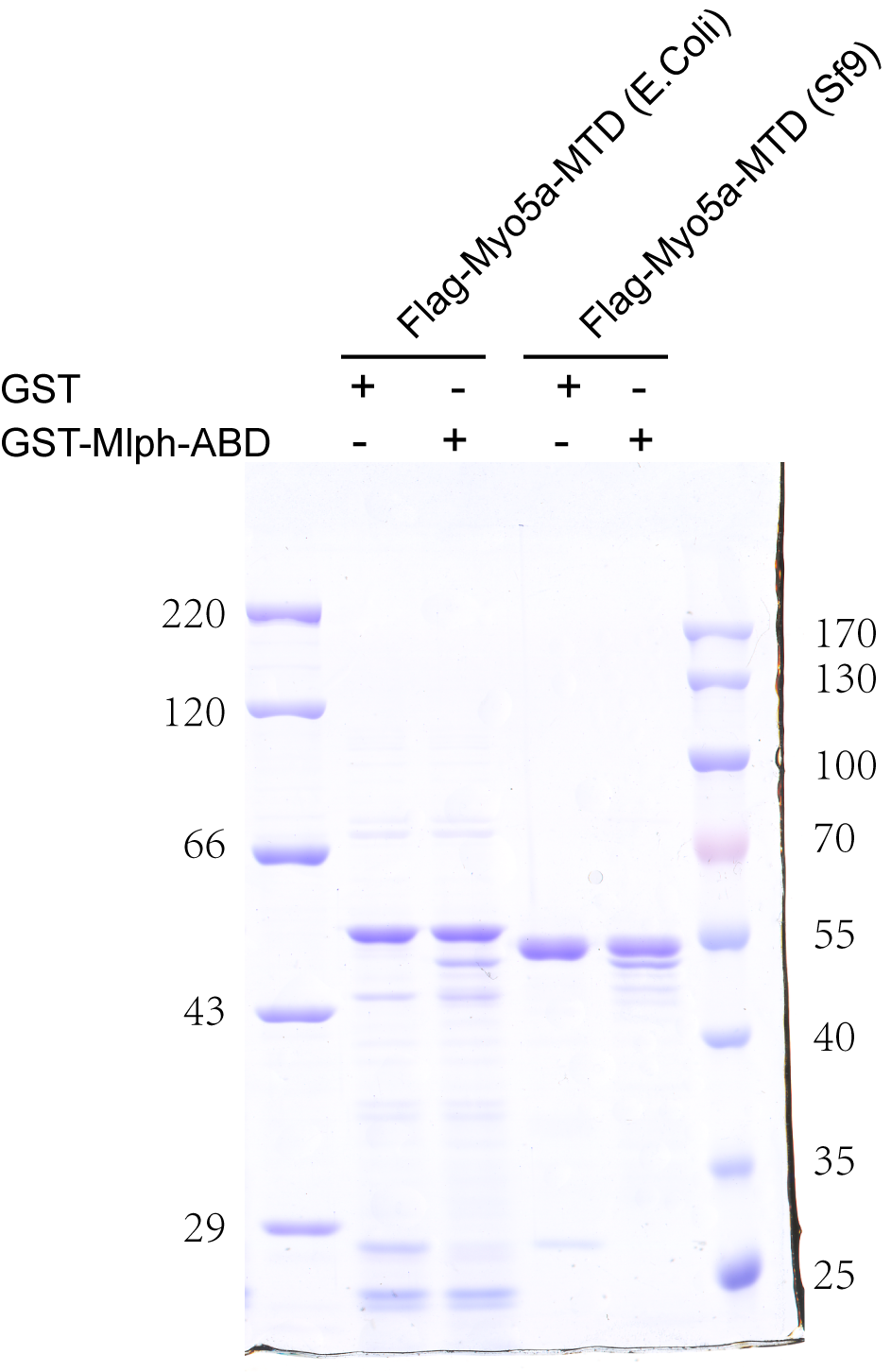

Supplement: Figure 2—figure supplement 1—source data 1. [file elife-93662-fig2-figsupp1-data1.zip › Figure 2-figure supplement Source data 1/Uncropped gels and blots for Figure 2 supplement A/Mlph-ABD pulldown Myo5a-MTD ecoli and sf9.tif]

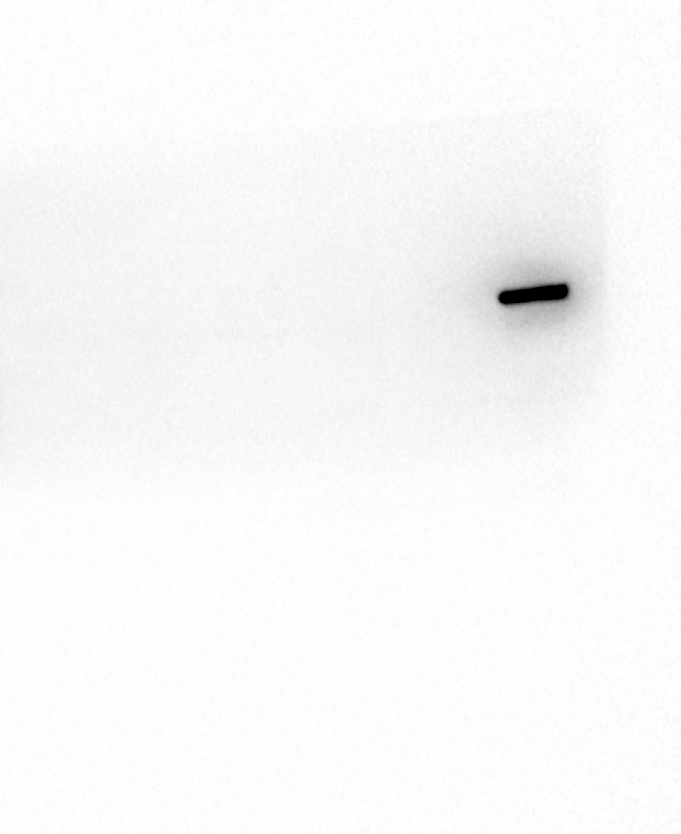

Supplement: Figure 2—figure supplement 1—source data 2. [file elife-93662-fig2-figsupp1-data2.zip › Figure 2-figure supplement Source data 2/Original files for Figure 2 supplement B/Mlph-ABD pulldown Myo5a-MTD truncation wb.tif]

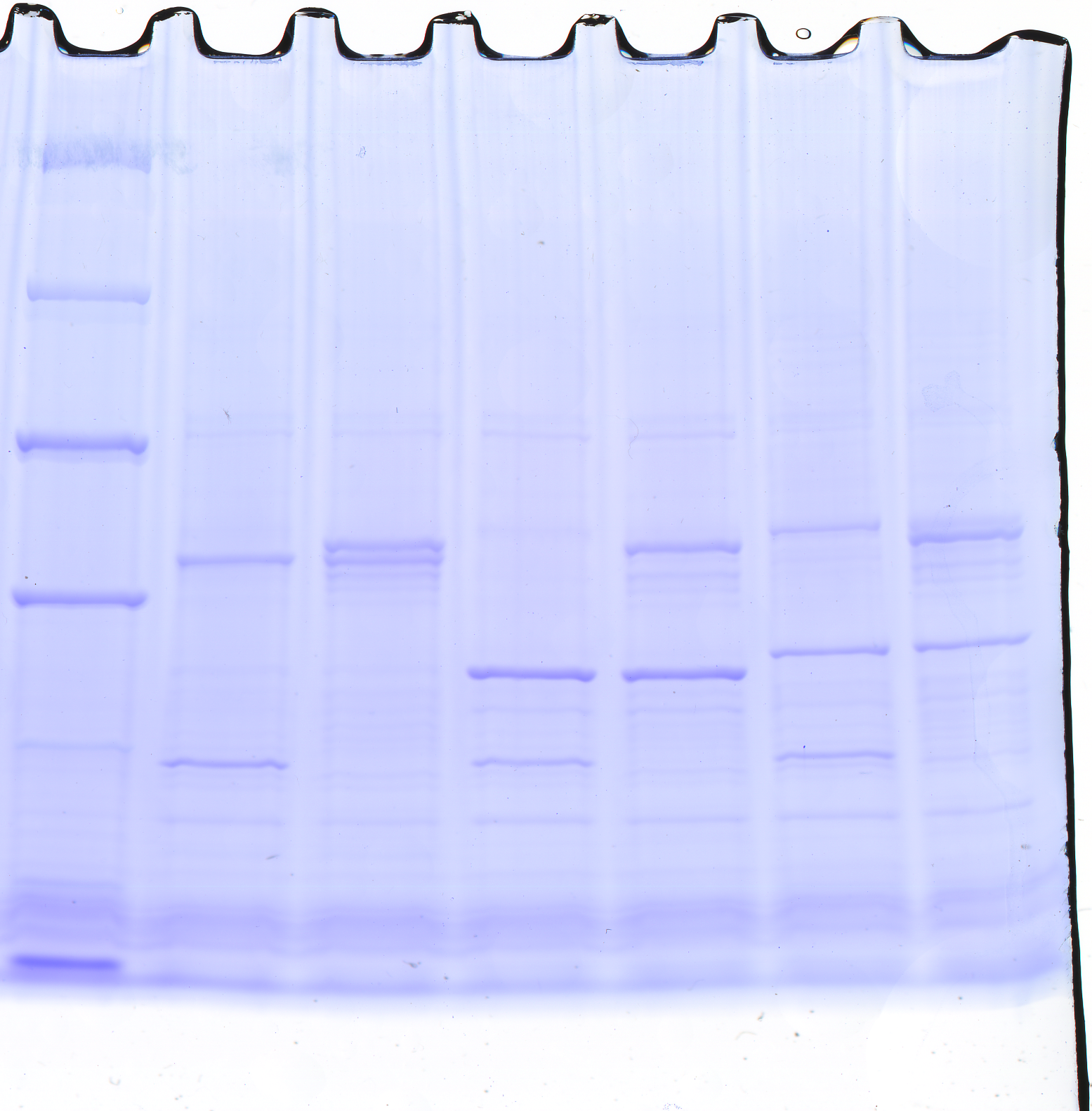

Supplement: Figure 2—figure supplement 1—source data 2. [file elife-93662-fig2-figsupp1-data2.zip › Figure 2-figure supplement Source data 2/Original files for Figure 2 supplement B/Mlph-ABD pulldown Myo5a-MTD truncation.tif]

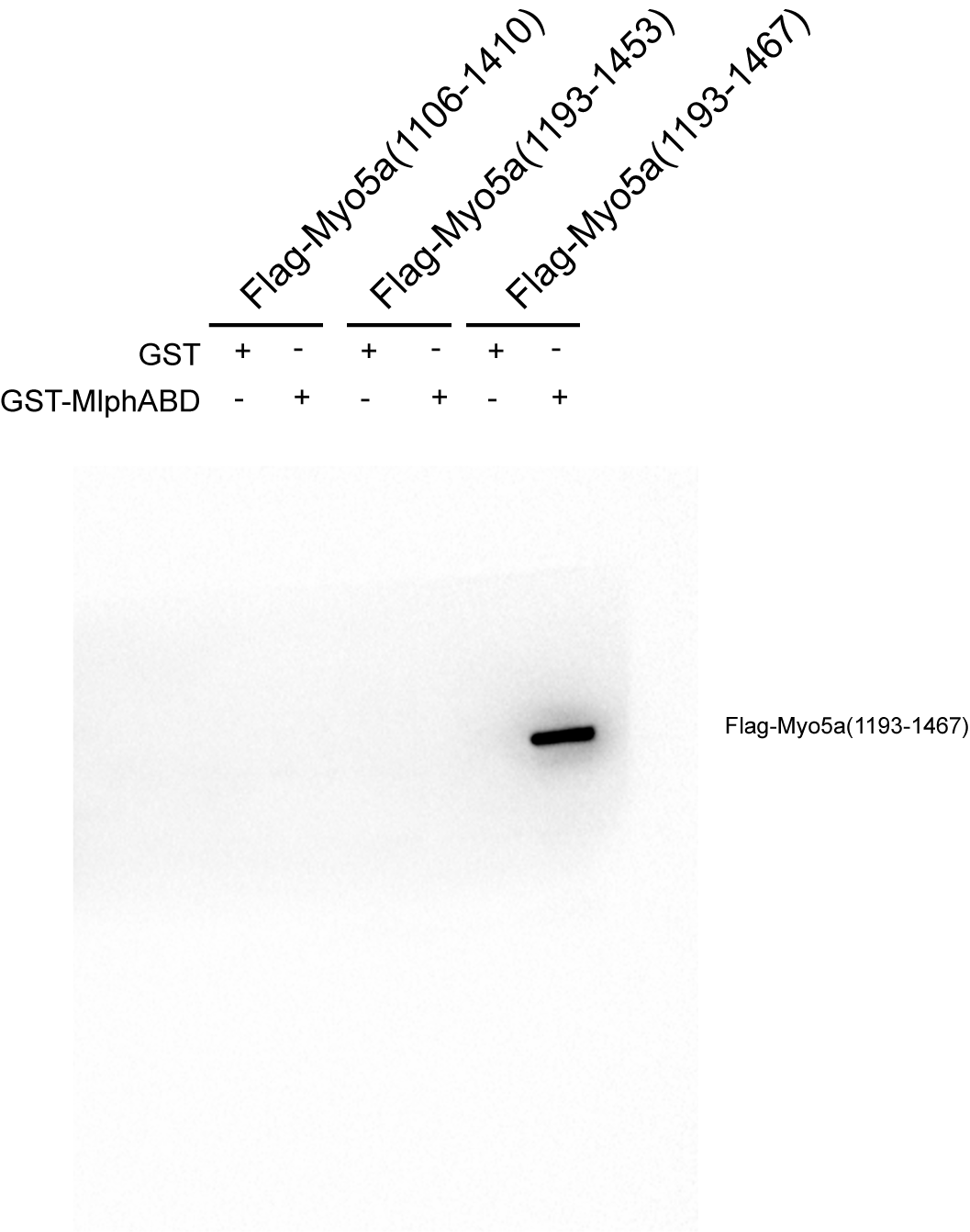

Supplement: Figure 2—figure supplement 1—source data 2. [file elife-93662-fig2-figsupp1-data2.zip › Figure 2-figure supplement Source data 2/Uncropped gels and blots for Figure 2 supplement B/Mlph-ABD pulldown Myo5a-MTD truncation wb.tif]

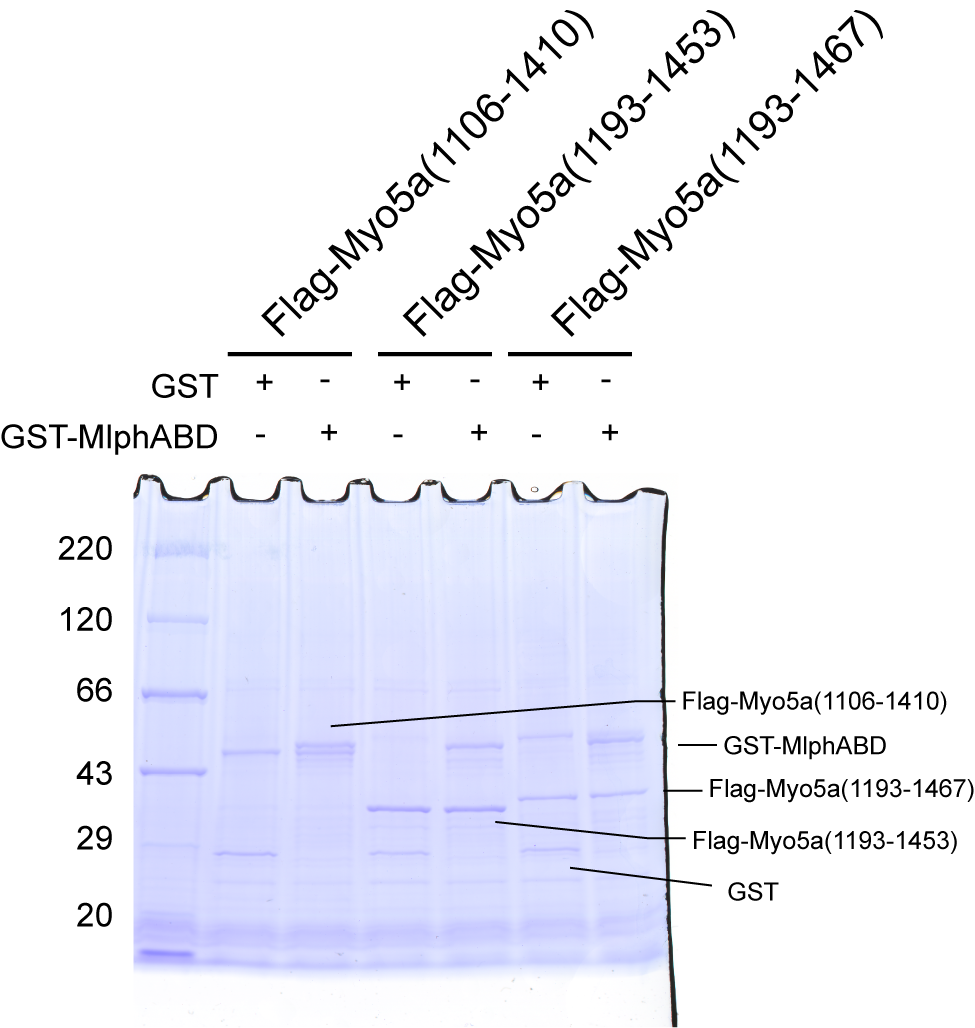

Supplement: Figure 2—figure supplement 1—source data 2. [file elife-93662-fig2-figsupp1-data2.zip › Figure 2-figure supplement Source data 2/Uncropped gels and blots for Figure 2 supplement B/Mlph-ABD pulldown Myo5a-MTD truncation.tif]

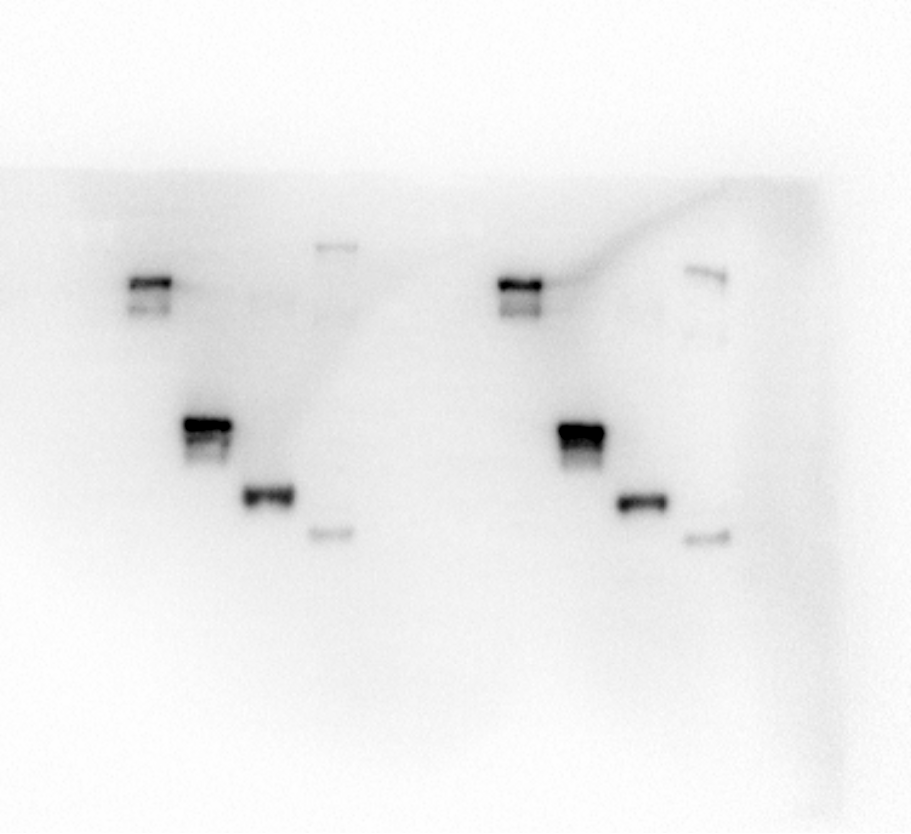

Supplement: Figure 2—figure supplement 1—source data 3. [file elife-93662-fig2-figsupp1-data3.zip › Figure 2-figure supplement Source data 3/Original files for Figure2 supplement C/Mlph-ABD pulldown Myo5a C truncation wb.tif]

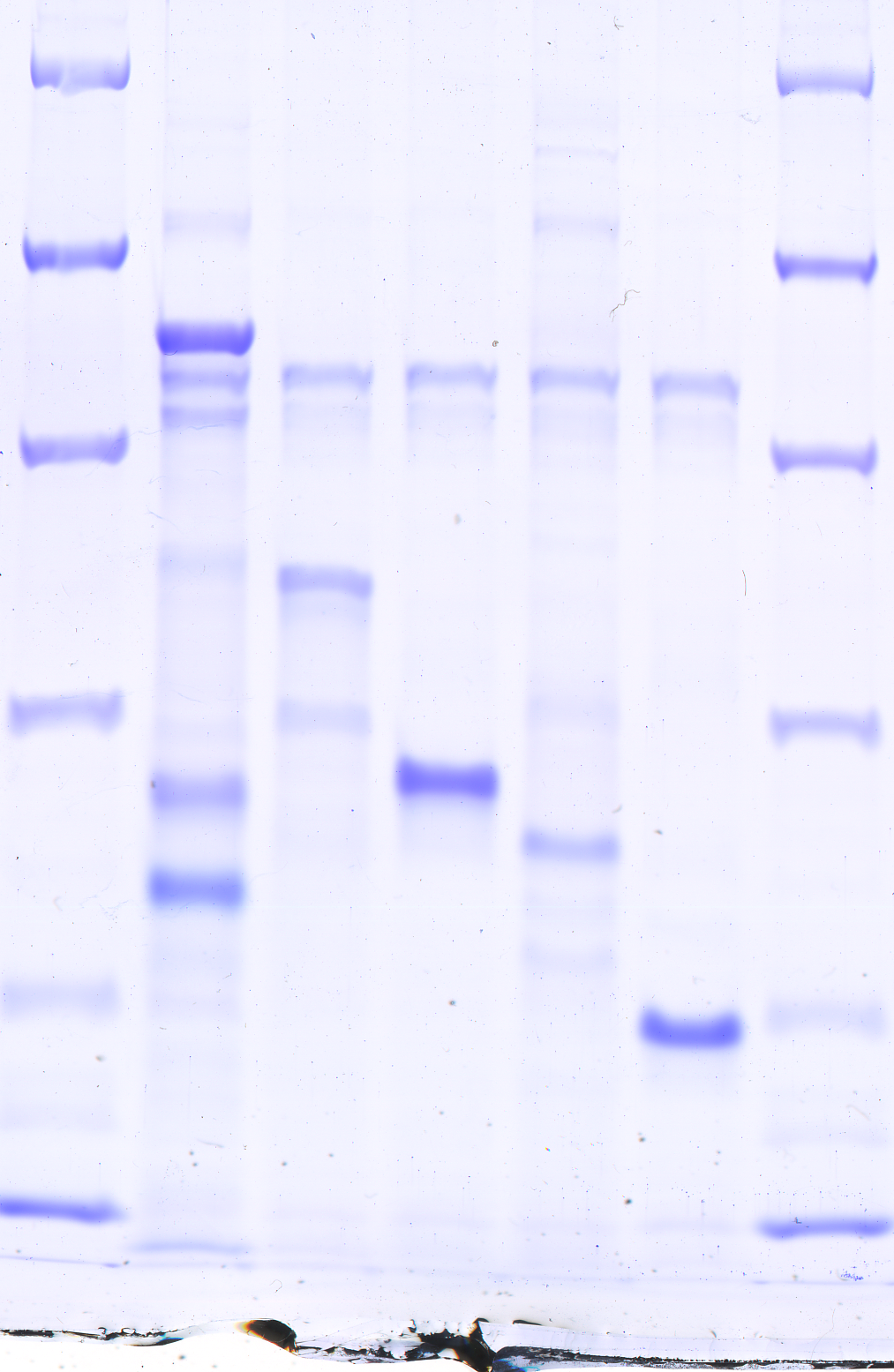

Supplement: Figure 2—figure supplement 1—source data 3. [file elife-93662-fig2-figsupp1-data3.zip › Figure 2-figure supplement Source data 3/Original files for Figure2 supplement C/Mlph-ABD pulldown Myo5a C truncation.tif]

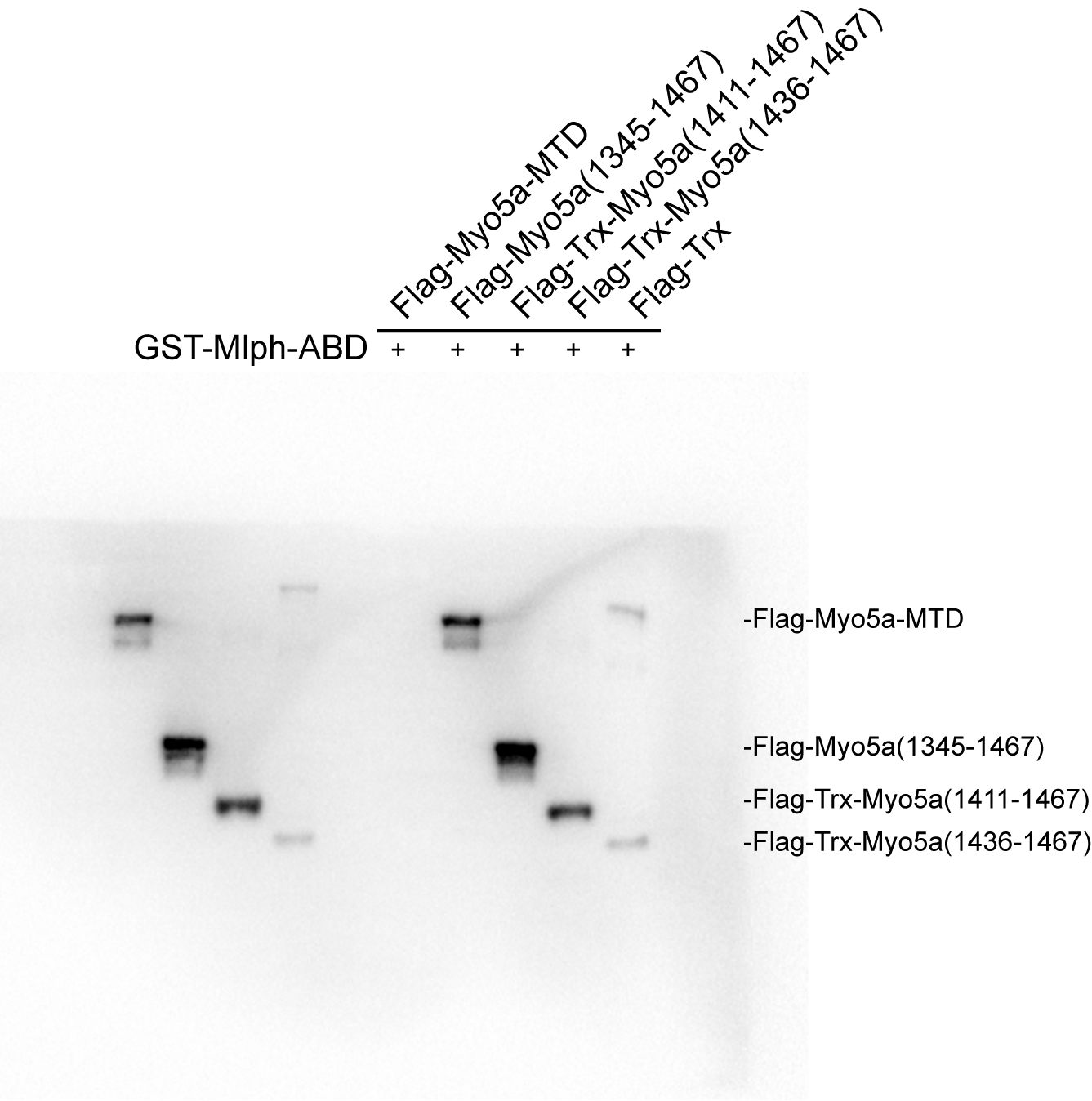

Supplement: Figure 2—figure supplement 1—source data 3. [file elife-93662-fig2-figsupp1-data3.zip › Figure 2-figure supplement Source data 3/Uncropped gels and blots for Figure2 supplement C/Mlph-ABD pulldown Myo5a C truncation wb.tif]

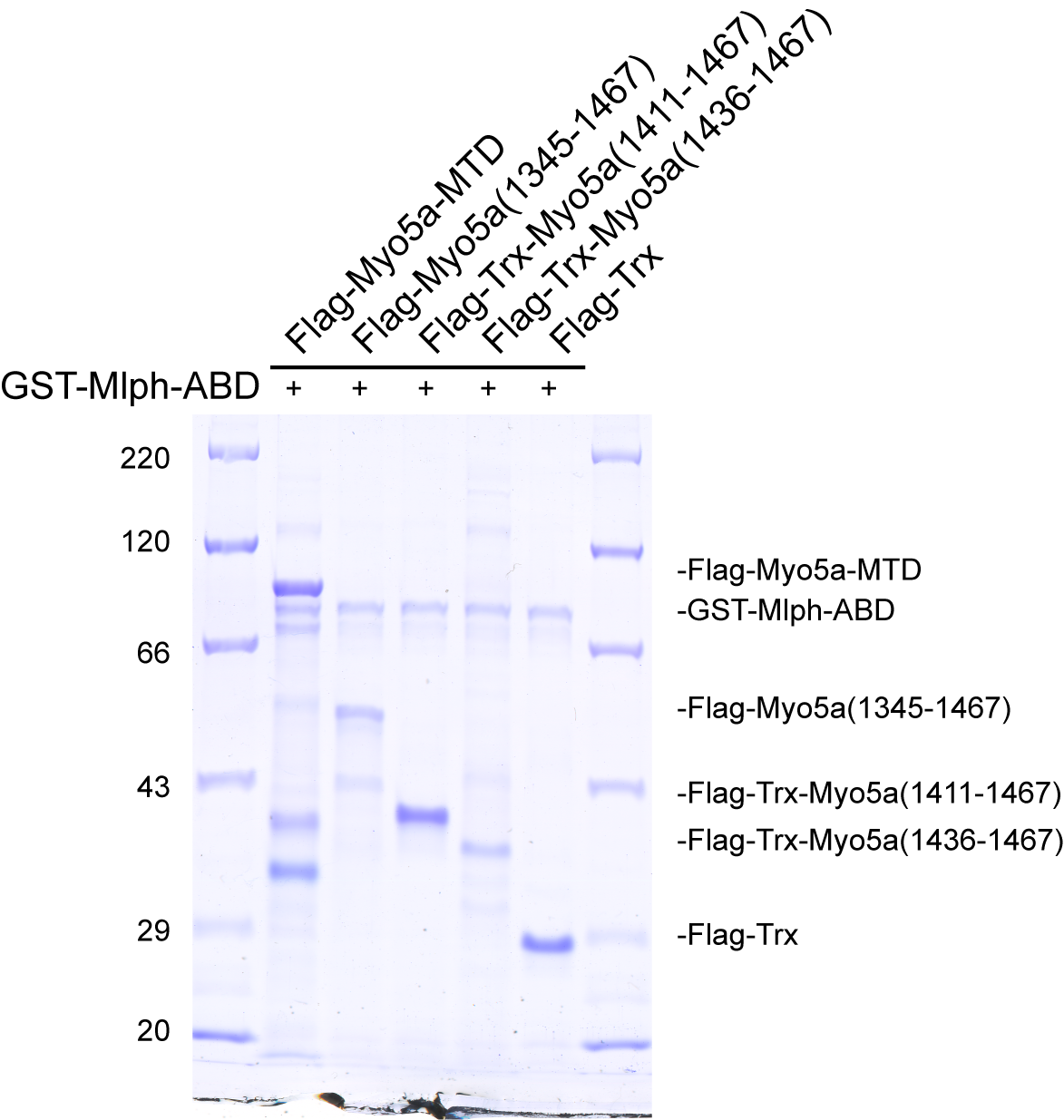

Supplement: Figure 2—figure supplement 1—source data 3. [file elife-93662-fig2-figsupp1-data3.zip › Figure 2-figure supplement Source data 3/Uncropped gels and blots for Figure2 supplement C/Mlph-ABD pulldown Myo5a C truncation.tif]

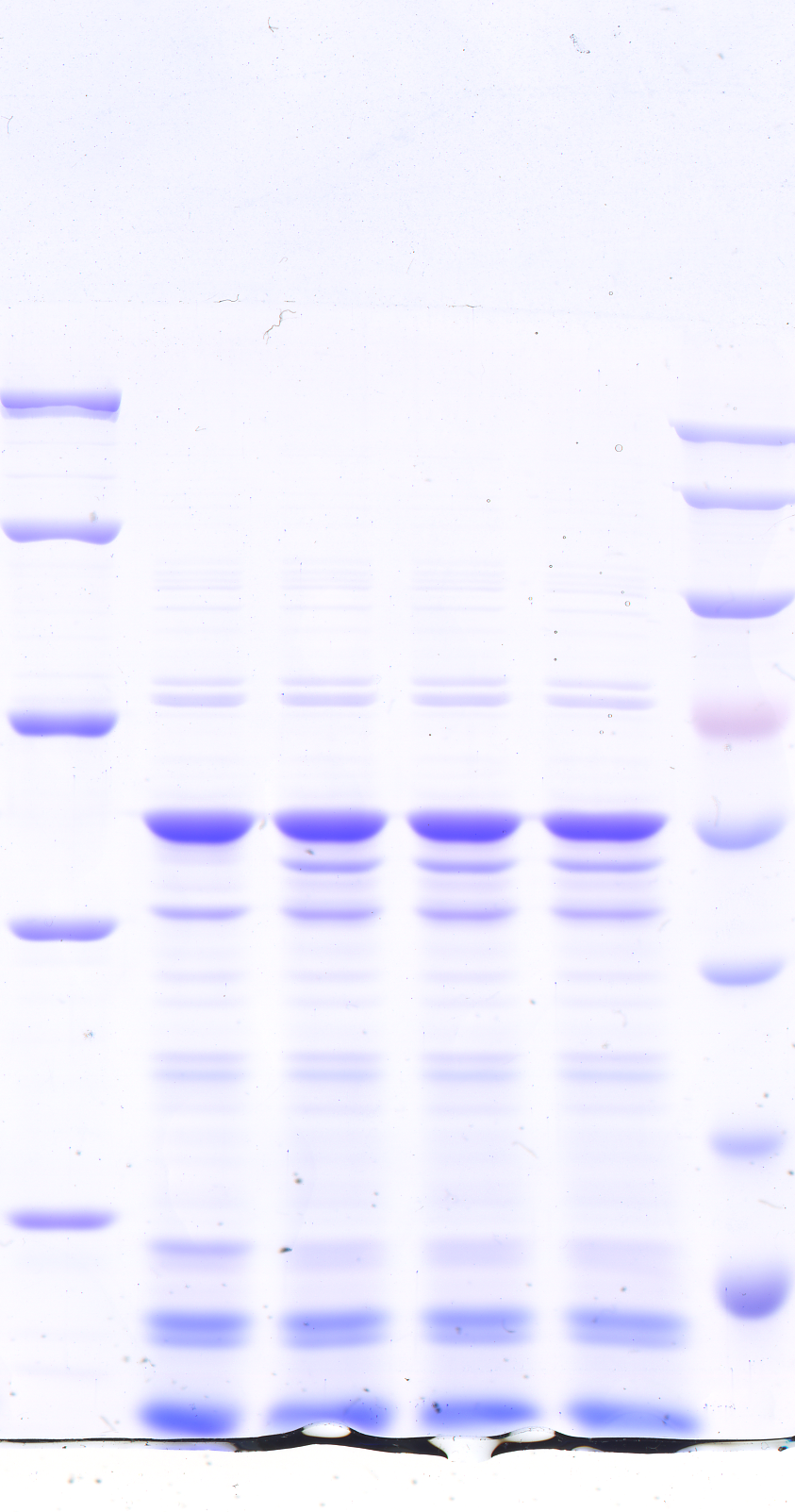

Supplement: Figure 3—source data 2. [file elife-93662-fig3-data2.zip › Figure 3-Source data 2/Original files for Figure 3A/Mlph-ABD pulldown Myo5a-MTD in diffreent NaCl strength Input.tif]

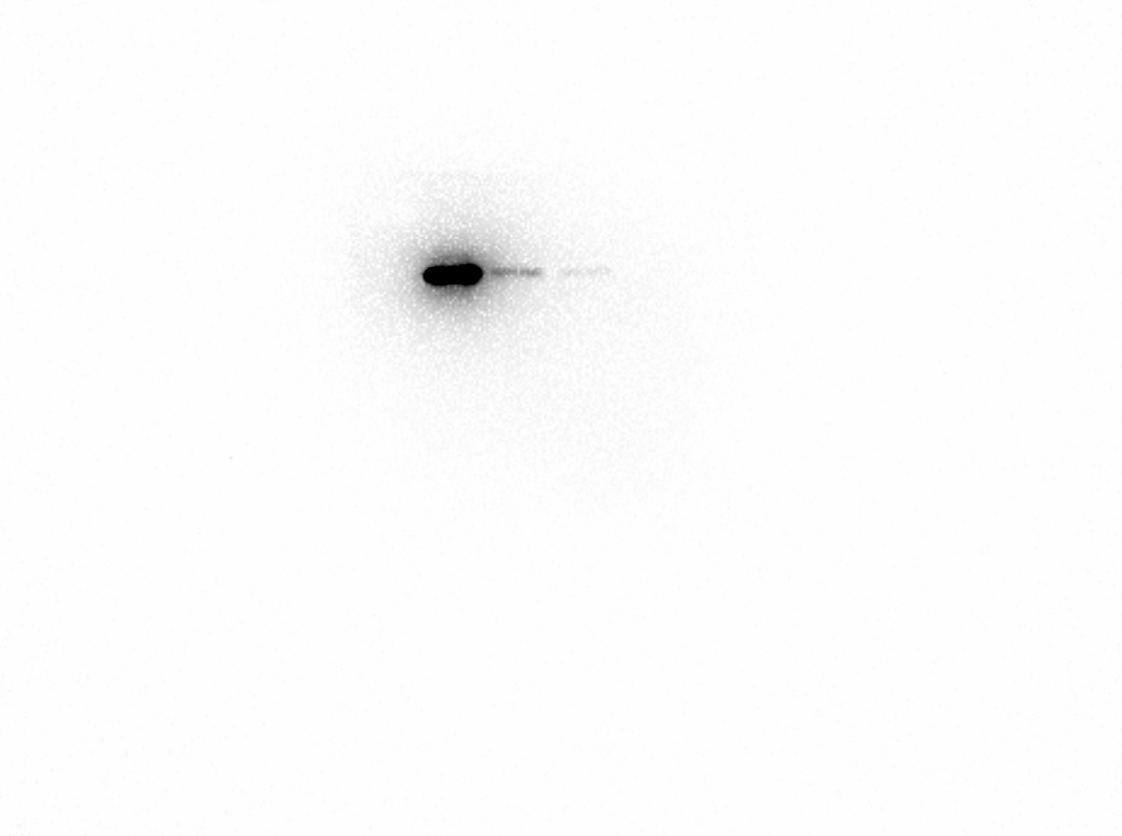

Supplement: Figure 3—source data 2. [file elife-93662-fig3-data2.zip › Figure 3-Source data 2/Original files for Figure 3A/Mlph-ABD pulldown Myo5a-MTD in diffreent NaCl strength wb.tif]

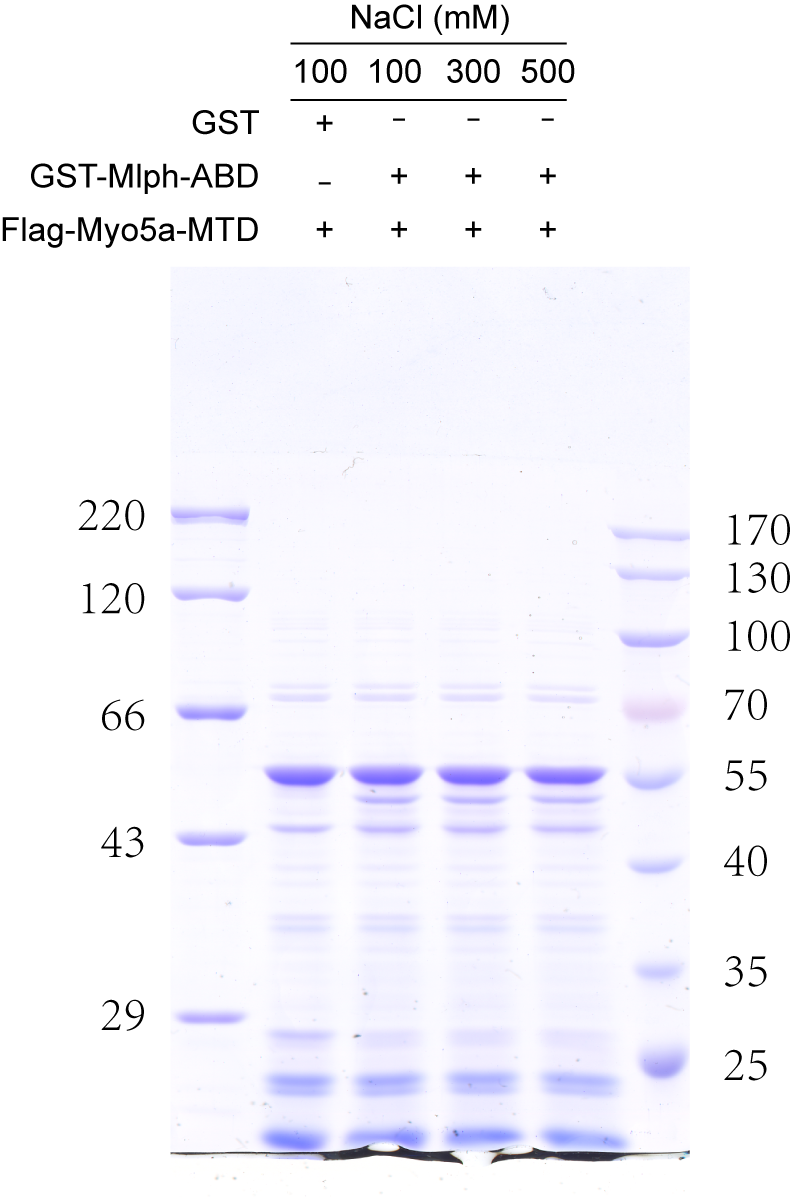

Supplement: Figure 3—source data 2. [file elife-93662-fig3-data2.zip › Figure 3-Source data 2/Uncropped gels and blots for Figure 3A/Mlph-ABD pulldown Myo5a-MTD in diffreent NaCl strength Input.tif]

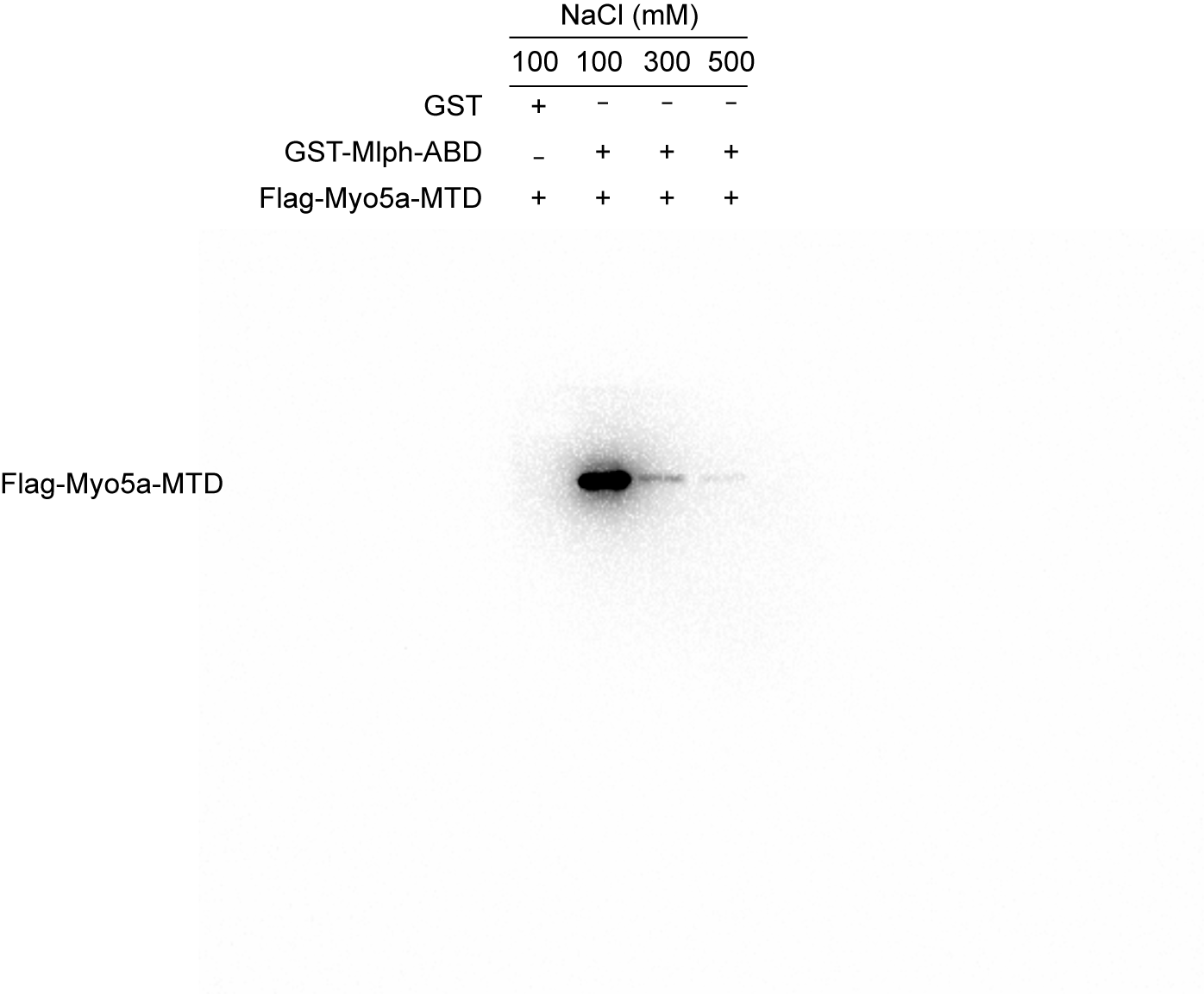

Supplement: Figure 3—source data 2. [file elife-93662-fig3-data2.zip › Figure 3-Source data 2/Uncropped gels and blots for Figure 3A/Mlph-ABD pulldown Myo5a-MTD in diffreent NaCl strength wb.tif]

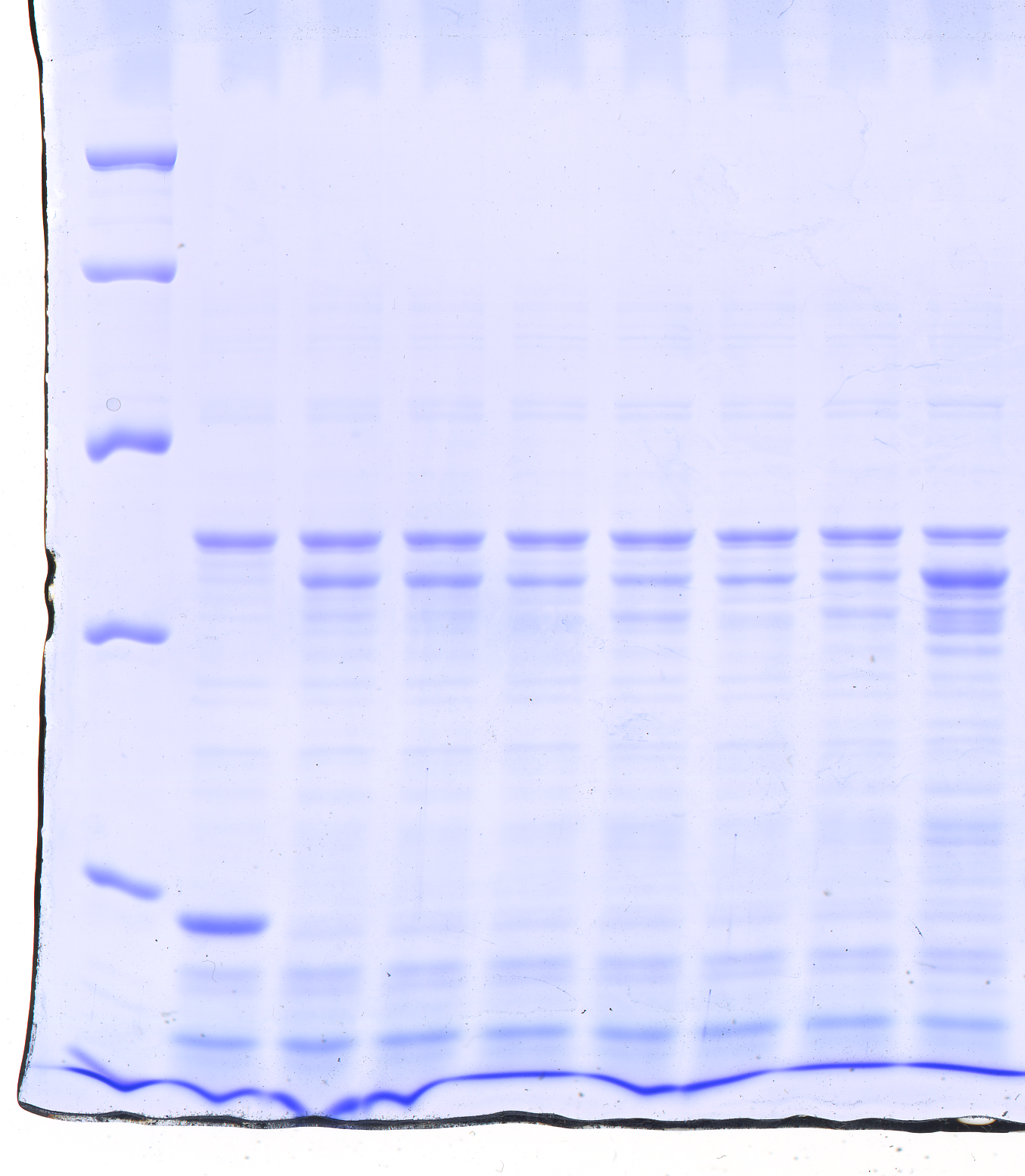

Supplement: Figure 3—source data 3. [file elife-93662-fig3-data3.zip › Figure 3-Source data 3/Original files for Figure 3C/Mlph-ABD and mutation pulldown Myo5a-MTD input.tif]

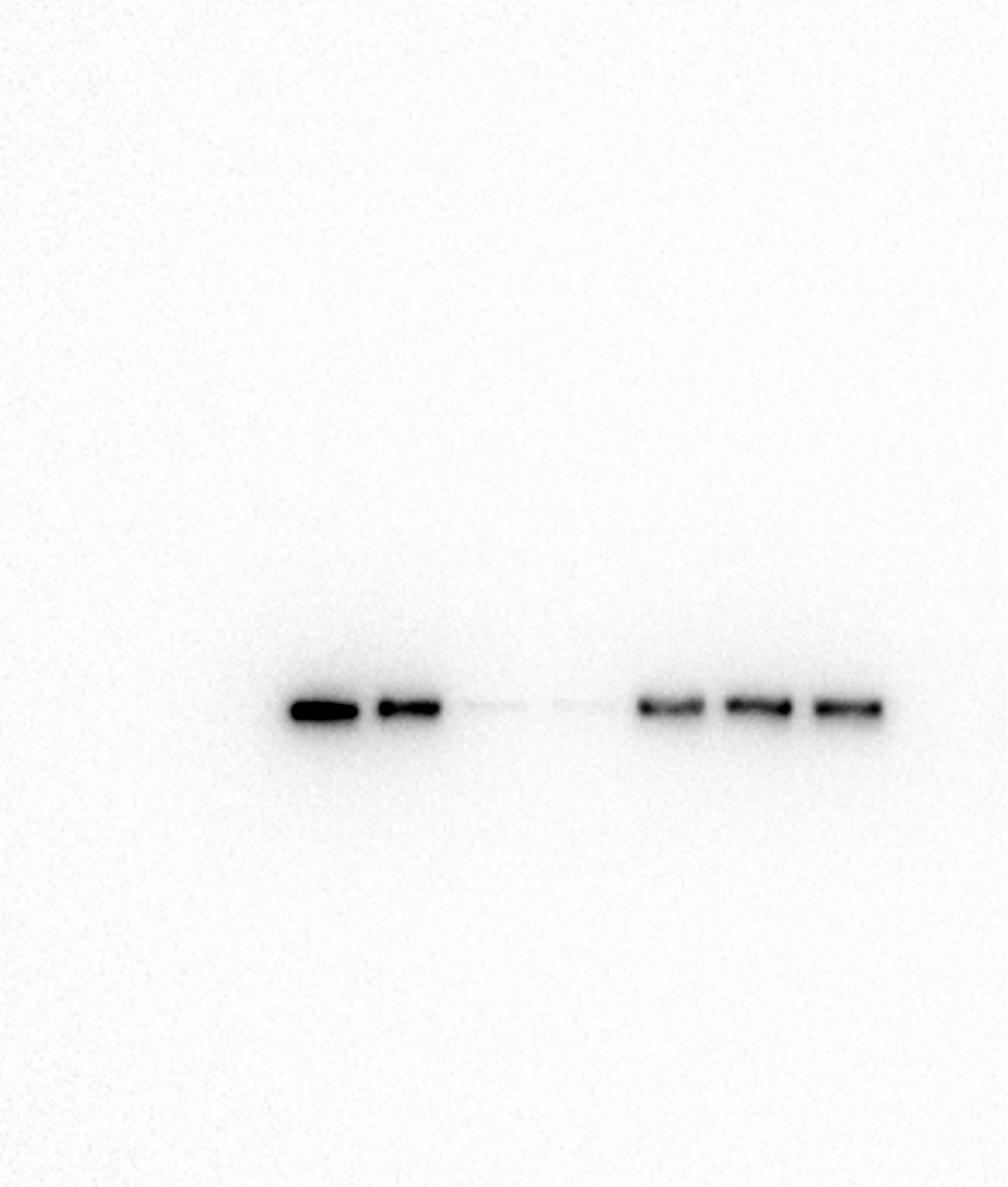

Supplement: Figure 3—source data 3. [file elife-93662-fig3-data3.zip › Figure 3-Source data 3/Original files for Figure 3C/Mlph-ABD and mutation pulldown Myo5a-MTD WB.tif]

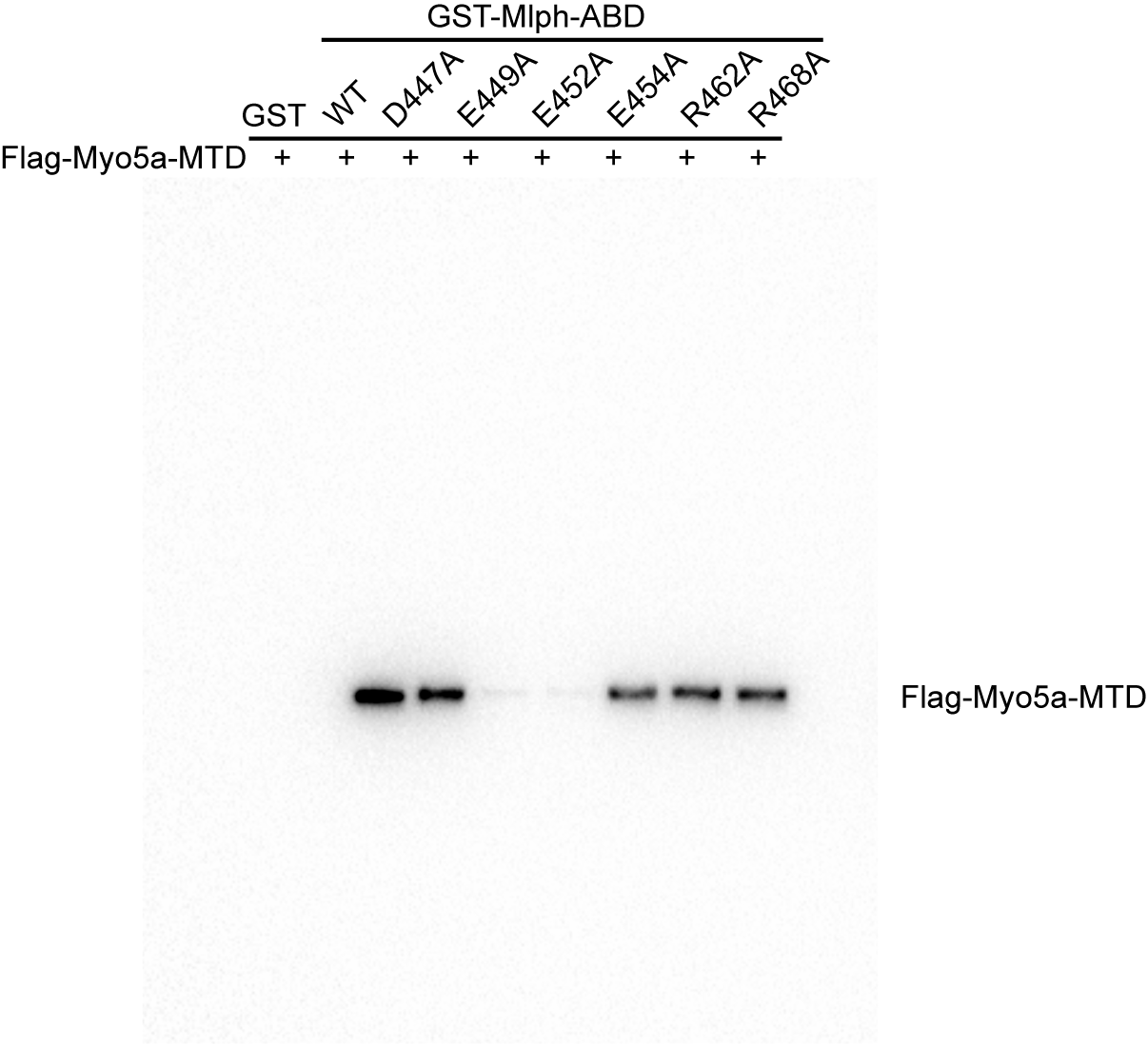

Supplement: Figure 3—source data 3. [file elife-93662-fig3-data3.zip › Figure 3-Source data 3/Uncropped gels and blots for Figure 3C/Mlph-ABD and mutation pulldown Myo5a-MTD WB.tif]

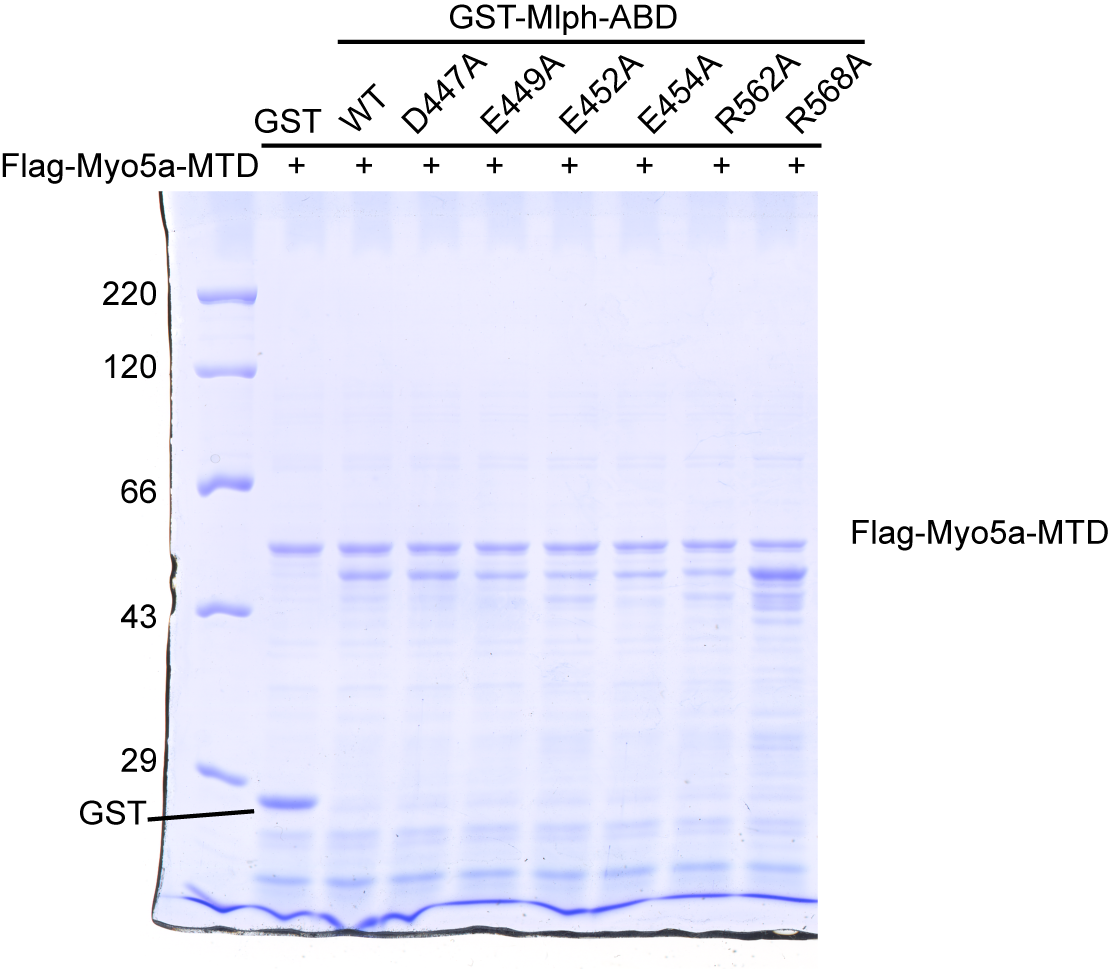

Supplement: Figure 3—source data 3. [file elife-93662-fig3-data3.zip › Figure 3-Source data 3/Uncropped gels and blots for Figure 3C/Mlph-ABD and mutation pulldown Myo5a-MTD.tif]

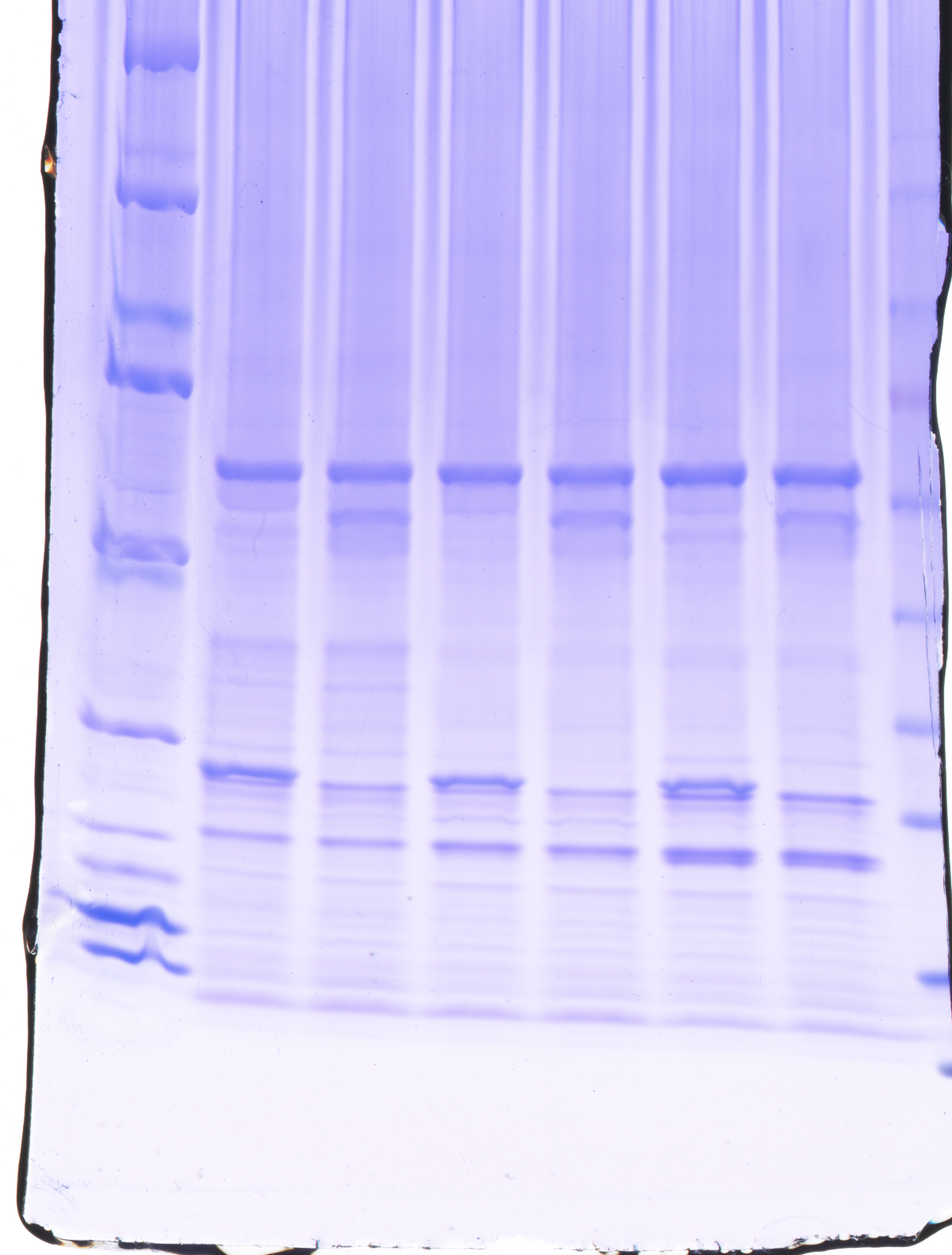

Supplement: Figure 3—source data 4. [file elife-93662-fig3-data4.zip › Figure 3-Source data 4/Original files for Figure 3E/Mlph-ABD pulldown Myo5a-MTD and mutation input.tif]

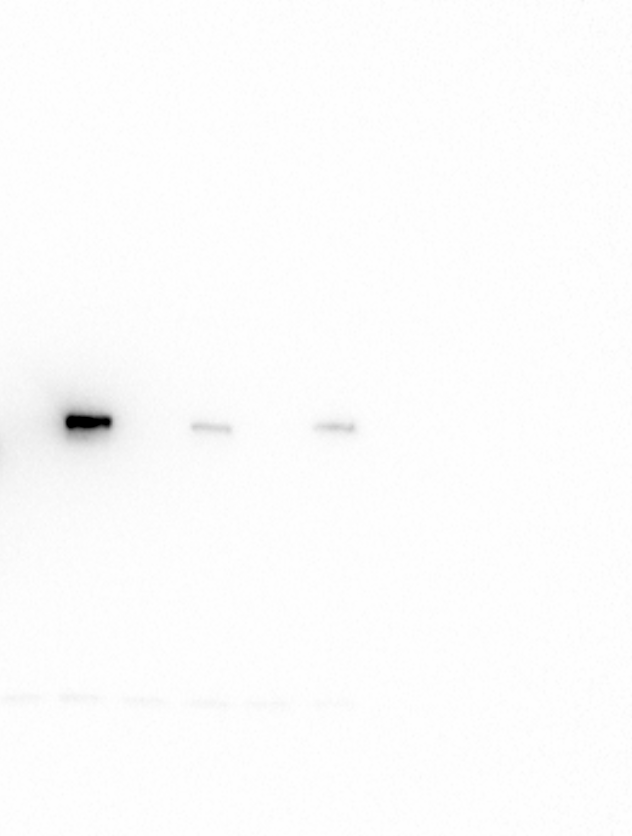

Supplement: Figure 3—source data 4. [file elife-93662-fig3-data4.zip › Figure 3-Source data 4/Original files for Figure 3E/Mlph-ABD pulldown Myo5a-MTD and mutation WB.tif]

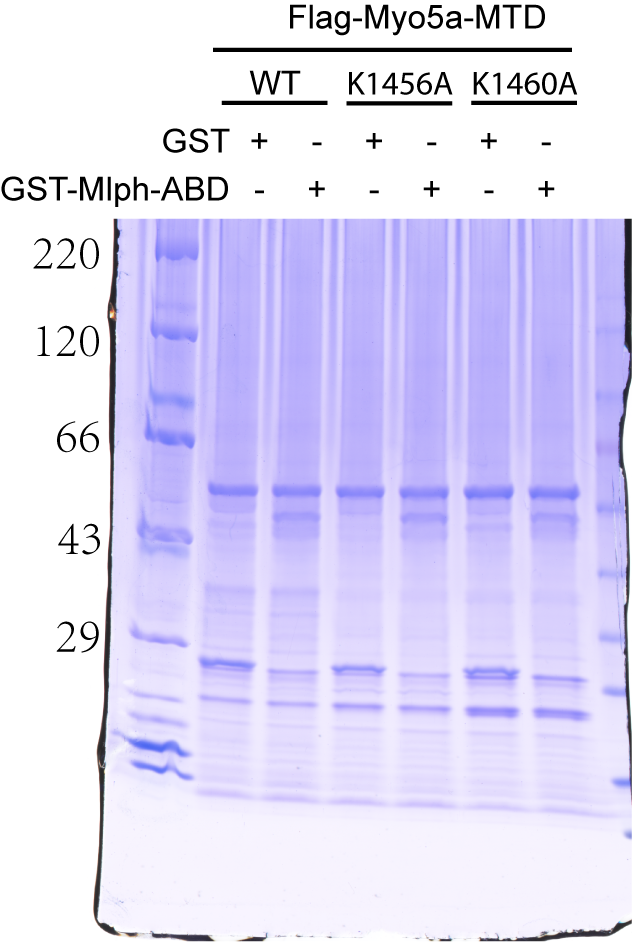

Supplement: Figure 3—source data 4. [file elife-93662-fig3-data4.zip › Figure 3-Source data 4/Uncropped gels and blots for Figure 3E/Mlph-ABD pulldown Myo5a-MTD and mutation input.tif]

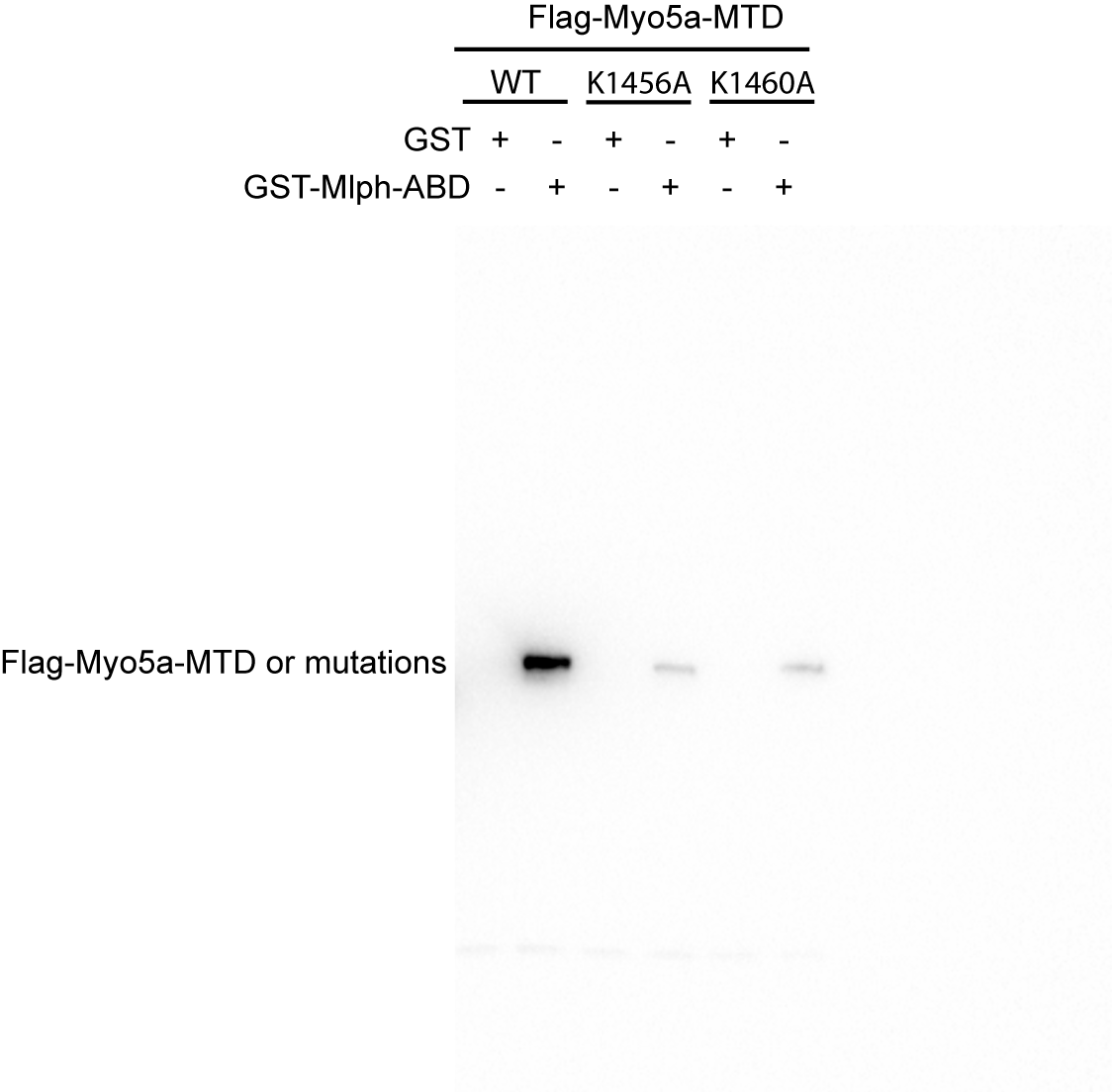

Supplement: Figure 3—source data 4. [file elife-93662-fig3-data4.zip › Figure 3-Source data 4/Uncropped gels and blots for Figure 3E/Mlph-ABD pulldown Myo5a-MTD and mutation WB.tif]

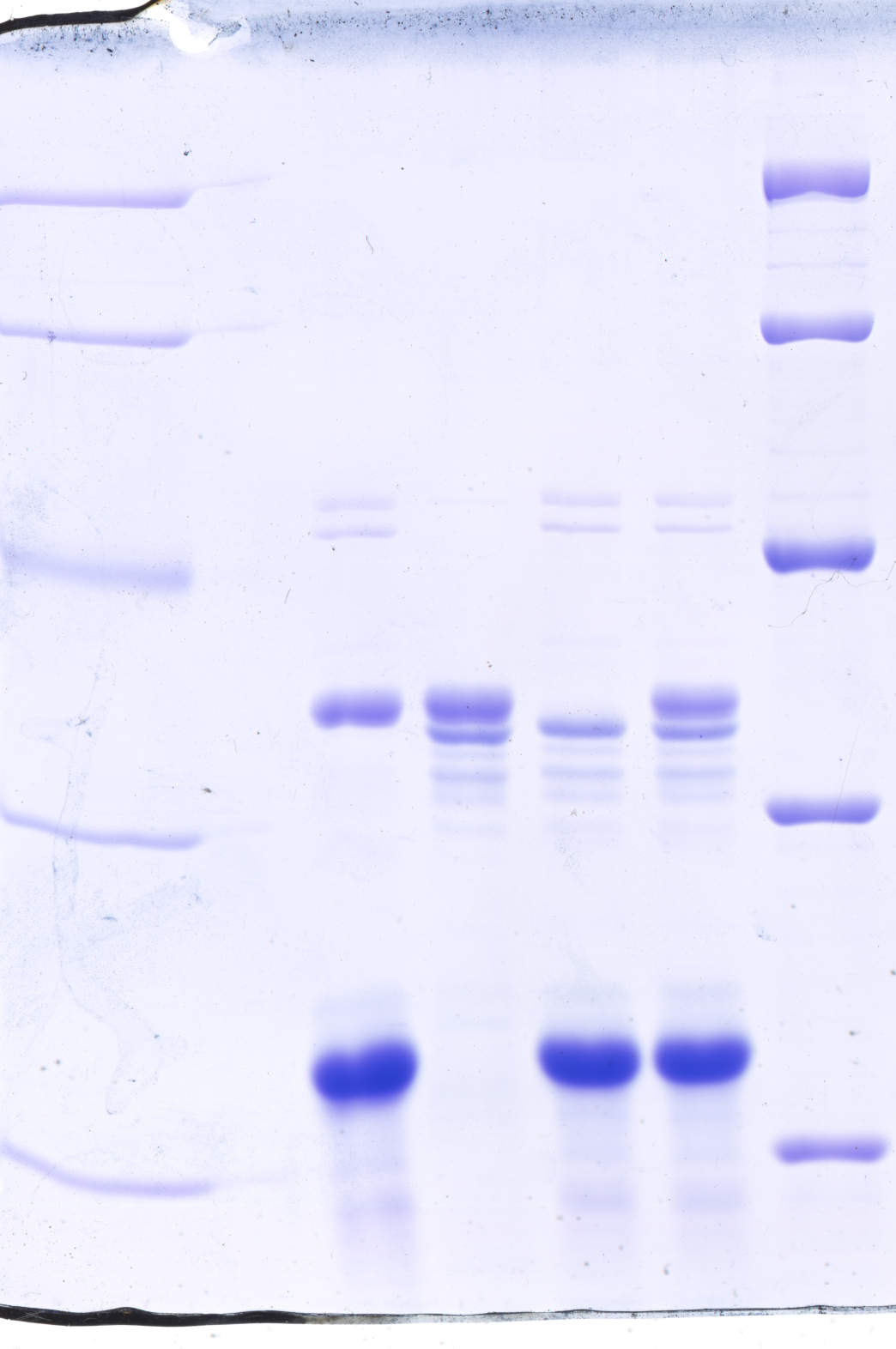

Supplement: Figure 4—source data 1. [file elife-93662-fig4-data1.zip › Figure 4-Source data 1/Original files for Figure 4A/M5a-MTD and Mlph-ABD EFBD input.tif]

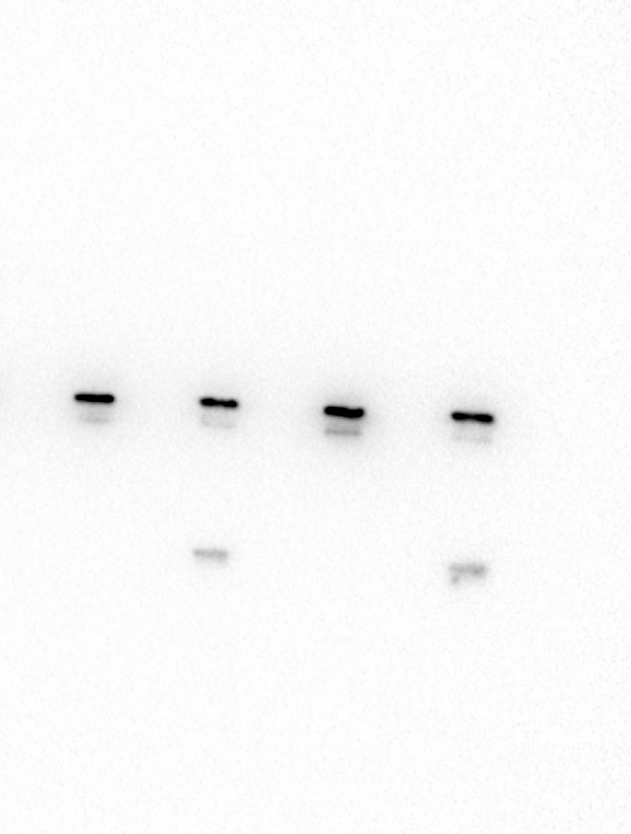

Supplement: Figure 4—source data 1. [file elife-93662-fig4-data1.zip › Figure 4-Source data 1/Original files for Figure 4A/M5a-MTD and Mlph-ABD EFBD wb.tif]

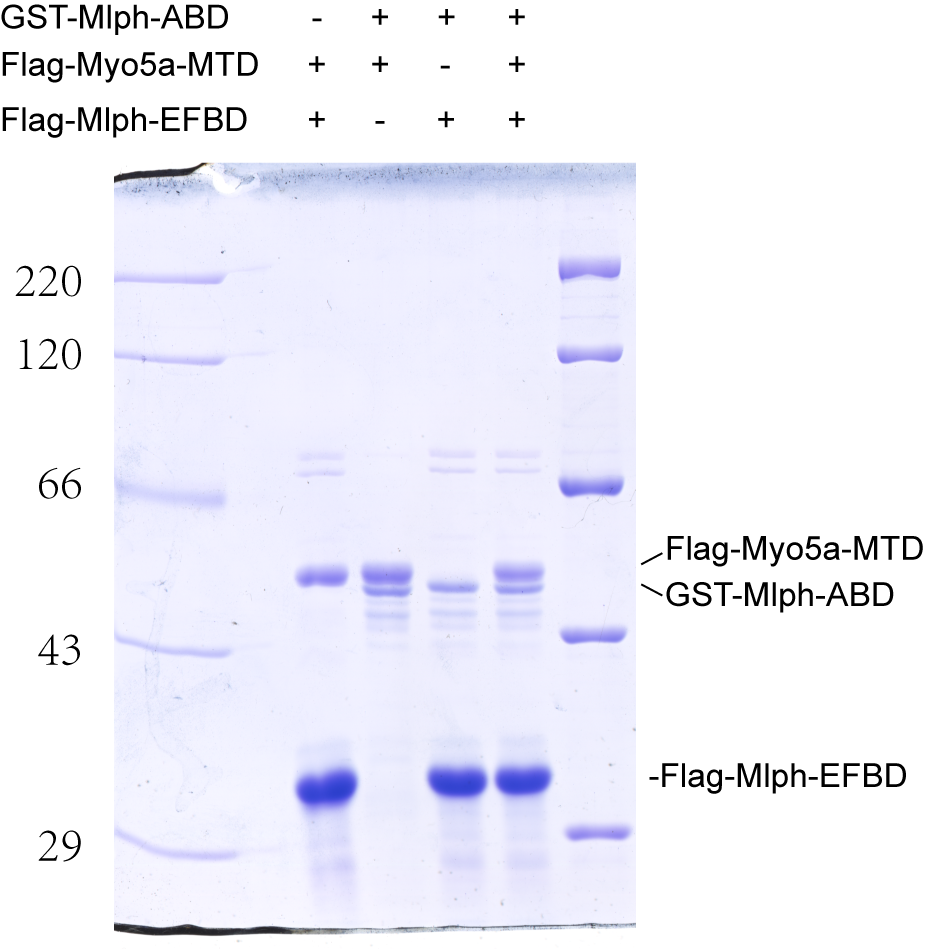

Supplement: Figure 4—source data 1. [file elife-93662-fig4-data1.zip › Figure 4-Source data 1/Uncropped gels and blots for Figure 4A/M5a-MTD and Mlph-ABD EFBD input.tif]

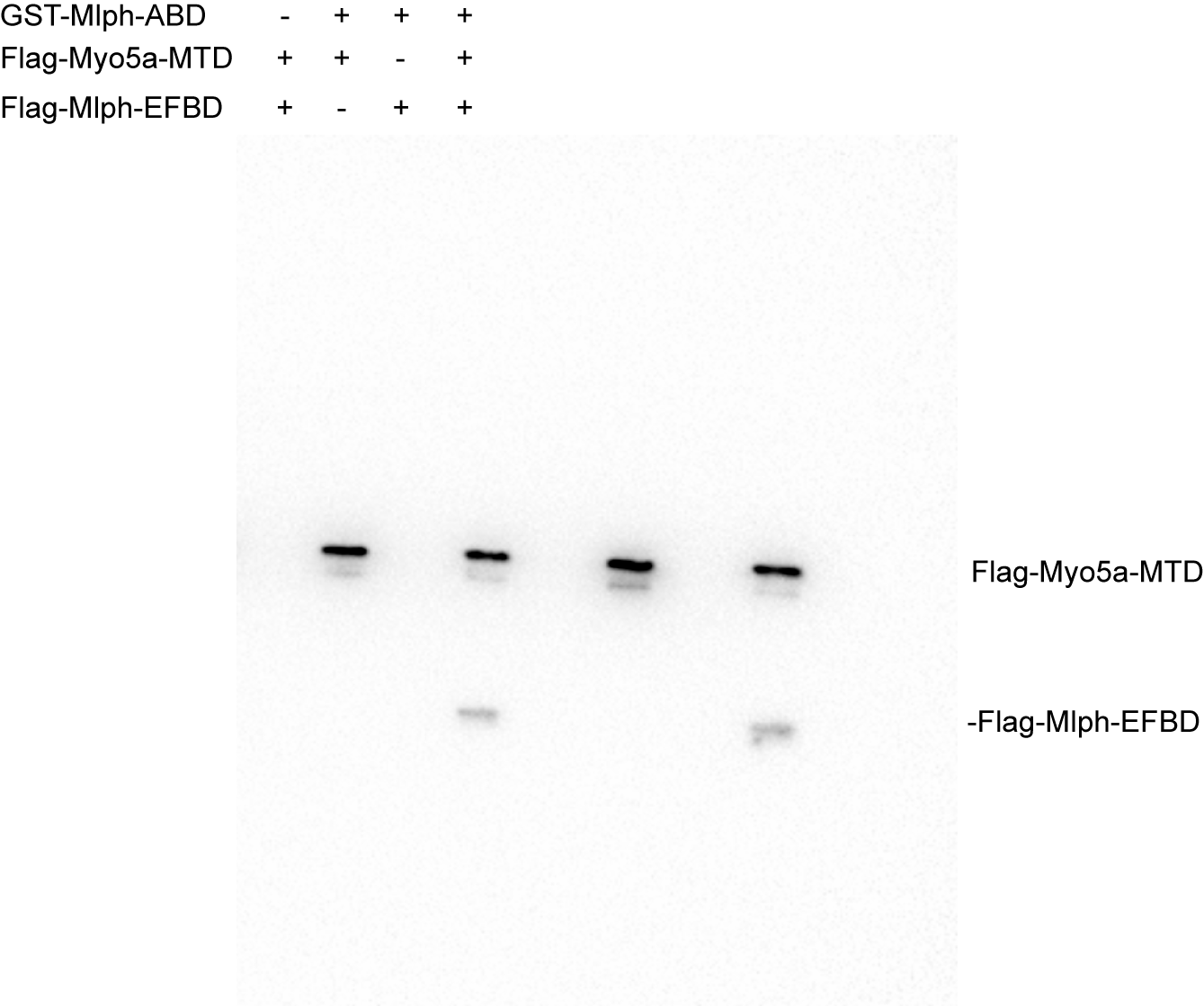

Supplement: Figure 4—source data 1. [file elife-93662-fig4-data1.zip › Figure 4-Source data 1/Uncropped gels and blots for Figure 4A/M5a-MTD and Mlph-ABD EFBD wb.tif]

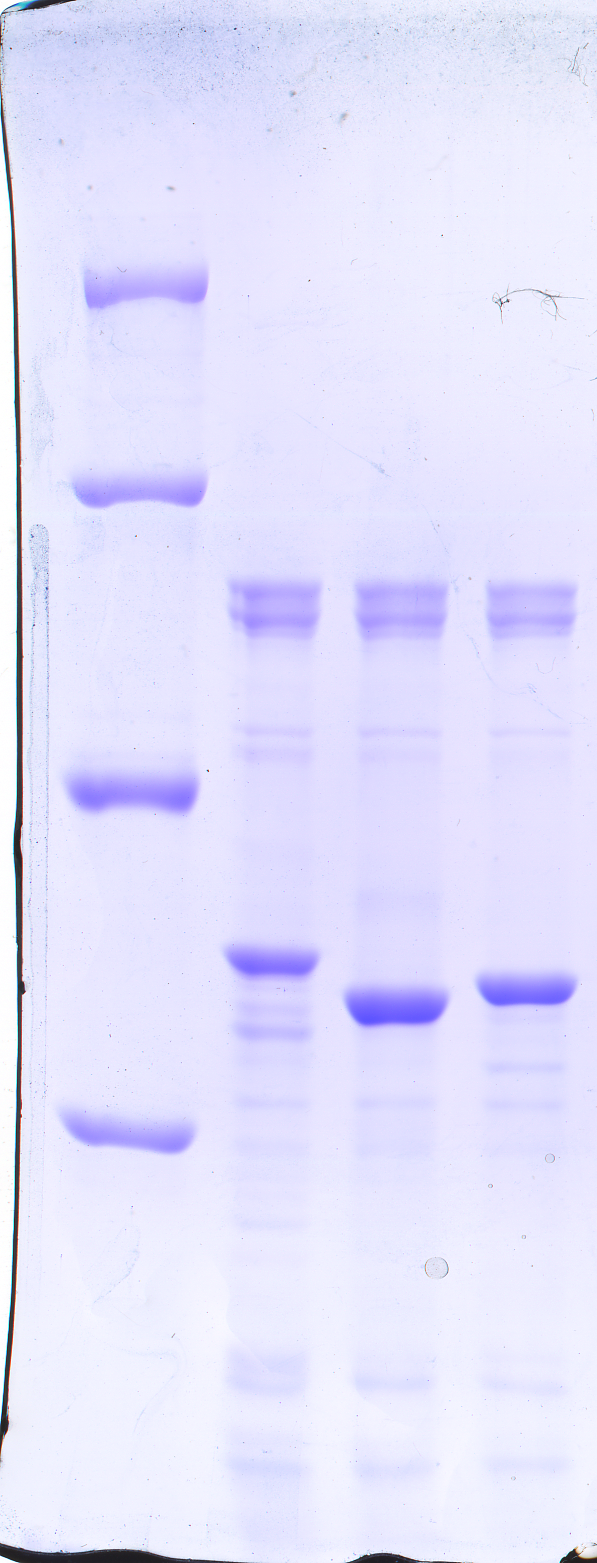

Supplement: Figure 4—source data 2. [file elife-93662-fig4-data2.zip › Figure 4-Source data 2/Original files for Figure 4B/Mlph-ΔABD pulldown Myo5a-MTD ΔF ΔG input.tif]

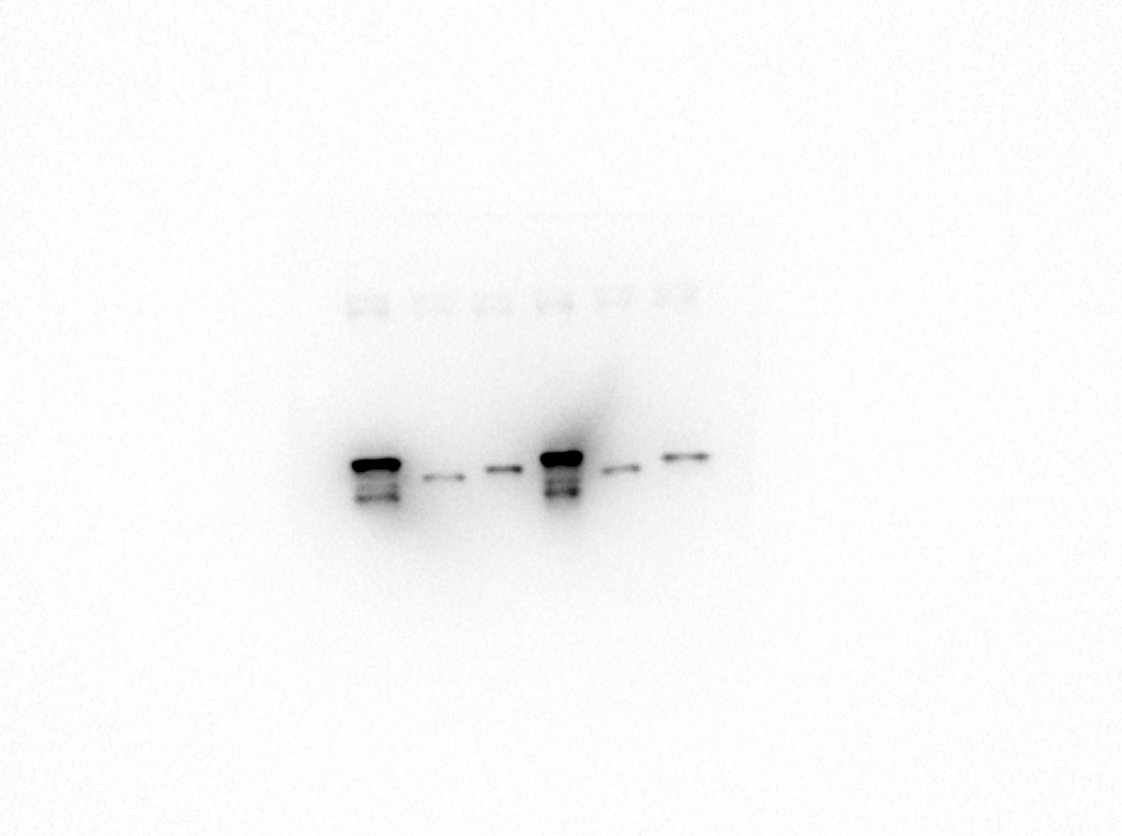

Supplement: Figure 4—source data 2. [file elife-93662-fig4-data2.zip › Figure 4-Source data 2/Original files for Figure 4B/Mlph-ΔABD pulldown Myo5a-MTD ΔF ΔG wb.tif]

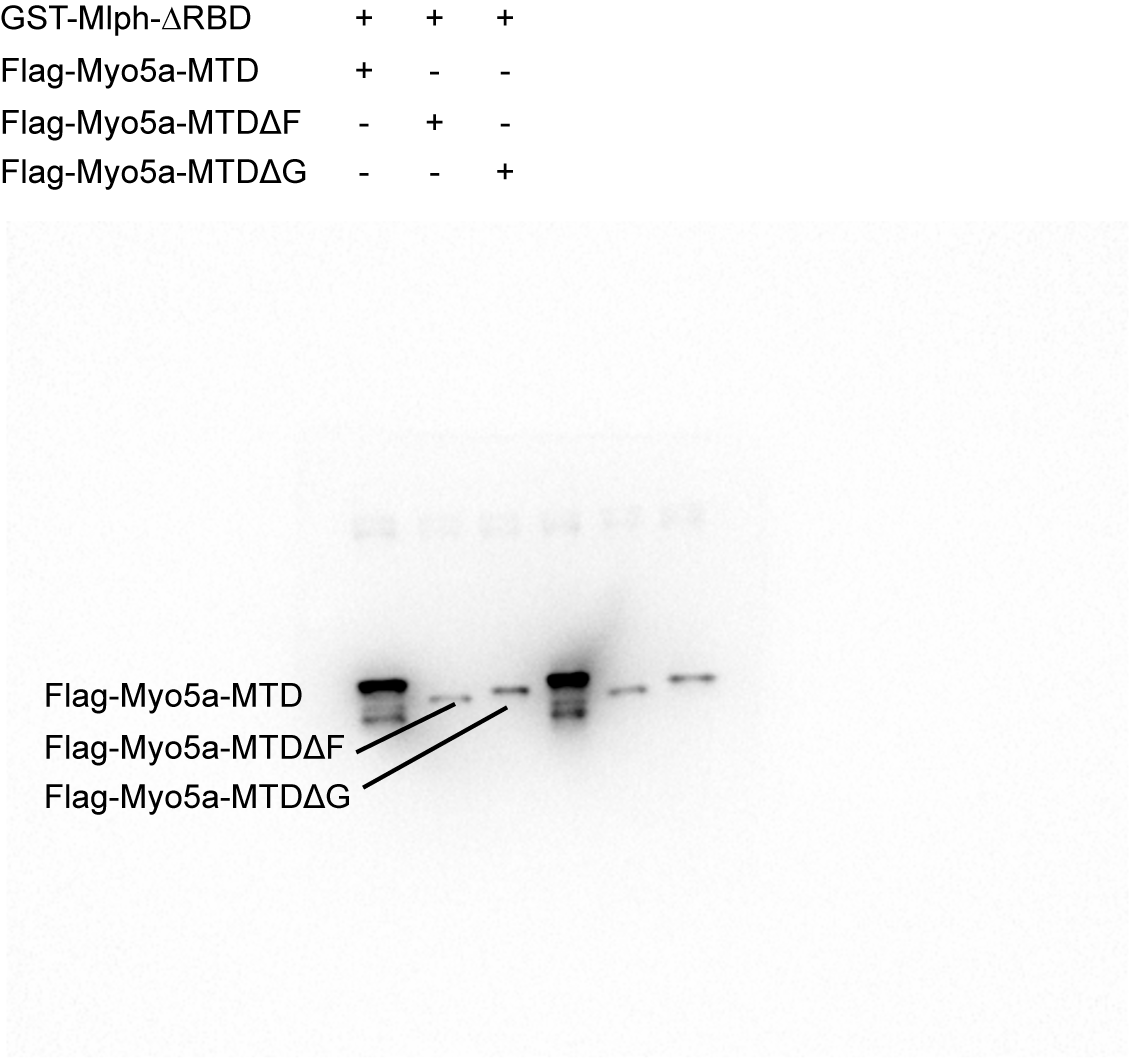

Supplement: Figure 4—source data 2. [file elife-93662-fig4-data2.zip › Figure 4-Source data 2/Uncropped gels and blots for Figure 4B/Mlph-ΔABD pulldown Myo5a-MTD ΔF ΔG WB.tif]

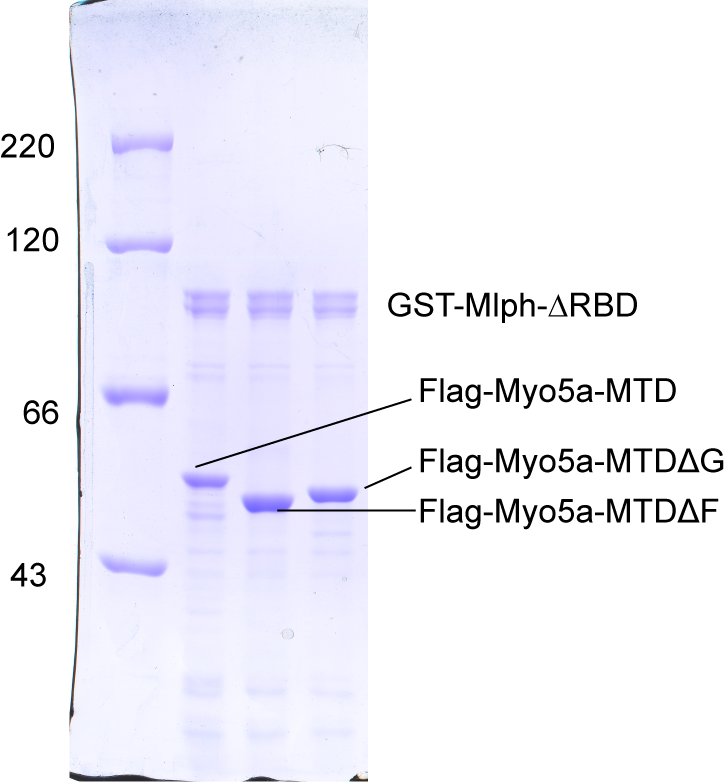

Supplement: Figure 4—source data 2. [file elife-93662-fig4-data2.zip › Figure 4-Source data 2/Uncropped gels and blots for Figure 4B/Mlph-ΔABDpulldown Myo5a-MTD ΔF ΔG.tif]

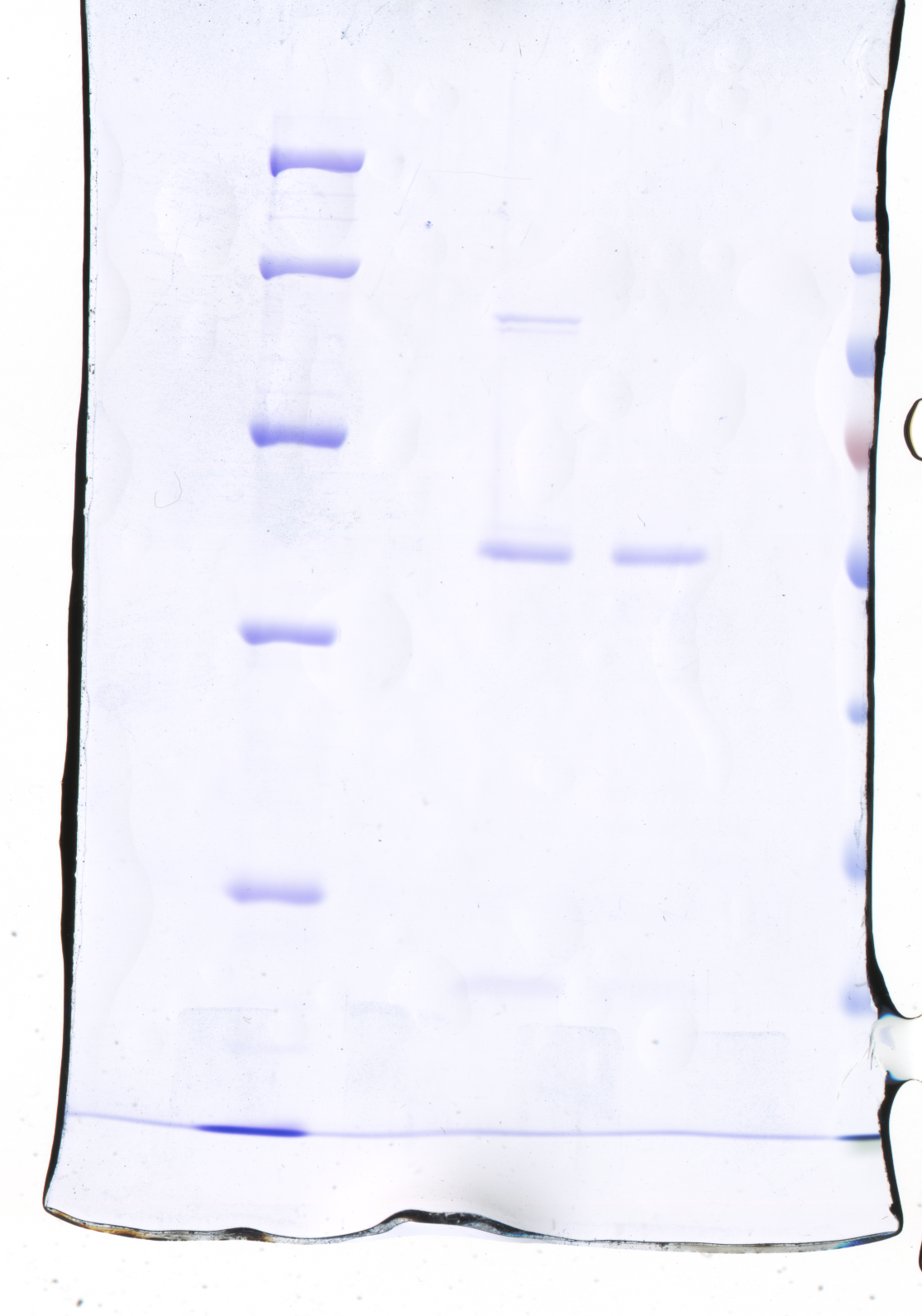

Supplement: Figure 4—source data 3. [file elife-93662-fig4-data3.zip › Figure 4-Source data 3/Original files for Figure 4C/Myo5a-MTD pulldown Mlph 148-590 and truncations CBB.tif]

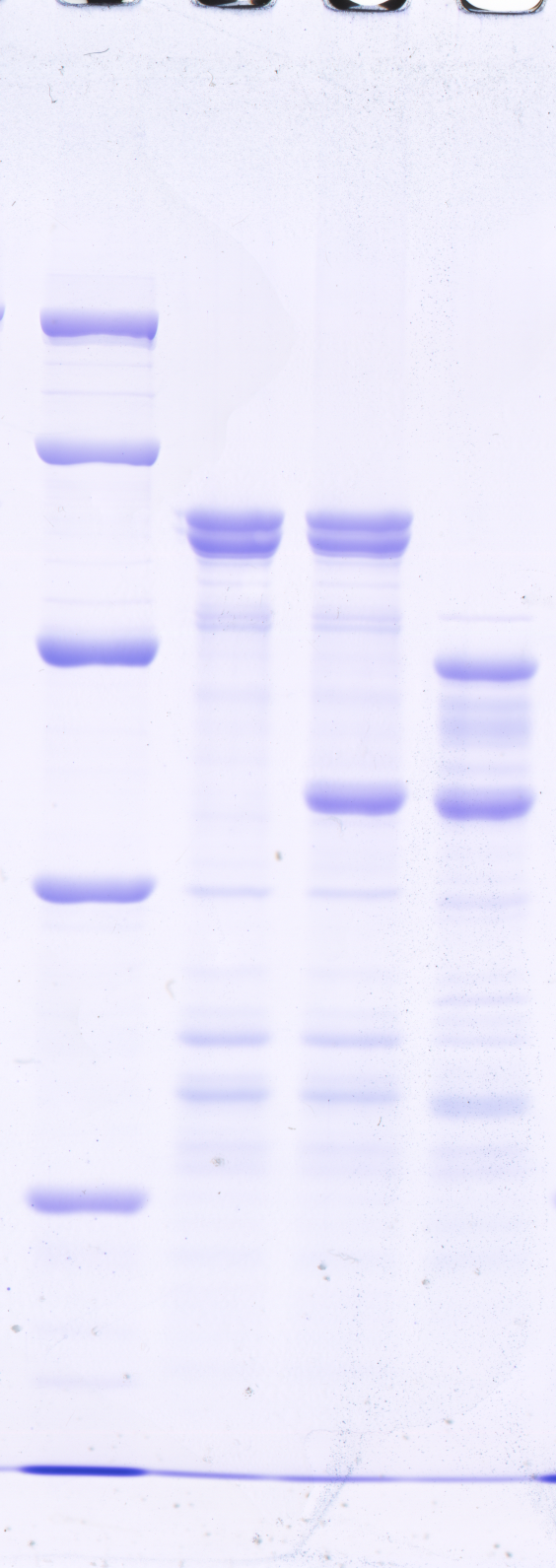

Supplement: Figure 4—source data 3. [file elife-93662-fig4-data3.zip › Figure 4-Source data 3/Original files for Figure 4C/Myo5a-MTD pulldown Mlph 148-590 and truncations input.tif]

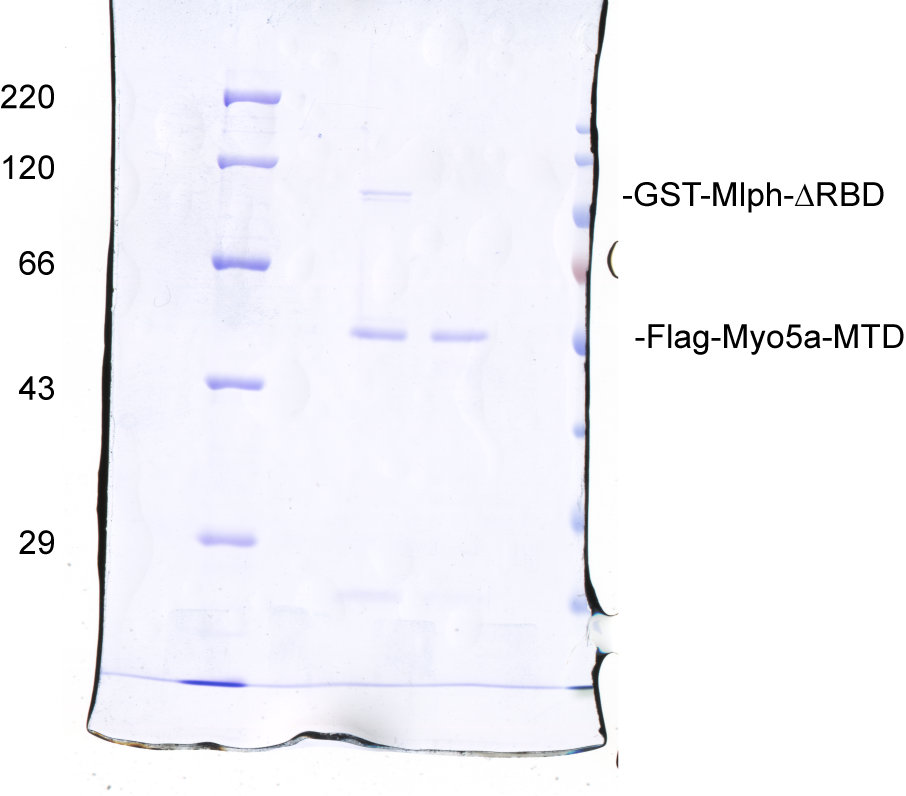

Supplement: Figure 4—source data 3. [file elife-93662-fig4-data3.zip › Figure 4-Source data 3/Uncropped gels for Figure 4C/Myo5a-MTD pulldown Mlph-ΔABD and truncations CBB.tif]

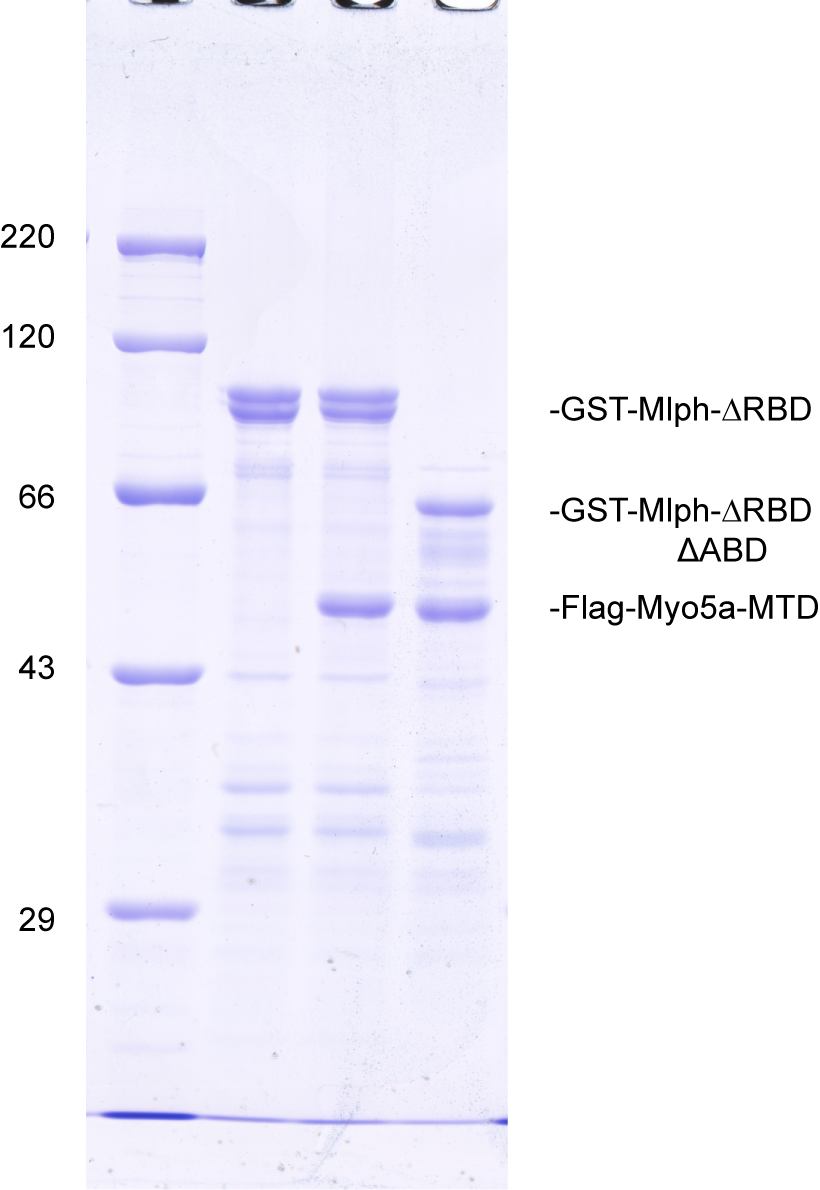

Supplement: Figure 4—source data 3. [file elife-93662-fig4-data3.zip › Figure 4-Source data 3/Uncropped gels for Figure 4C/Myo5a-MTD pulldown Mlph-ΔABD and truncations input.tif]

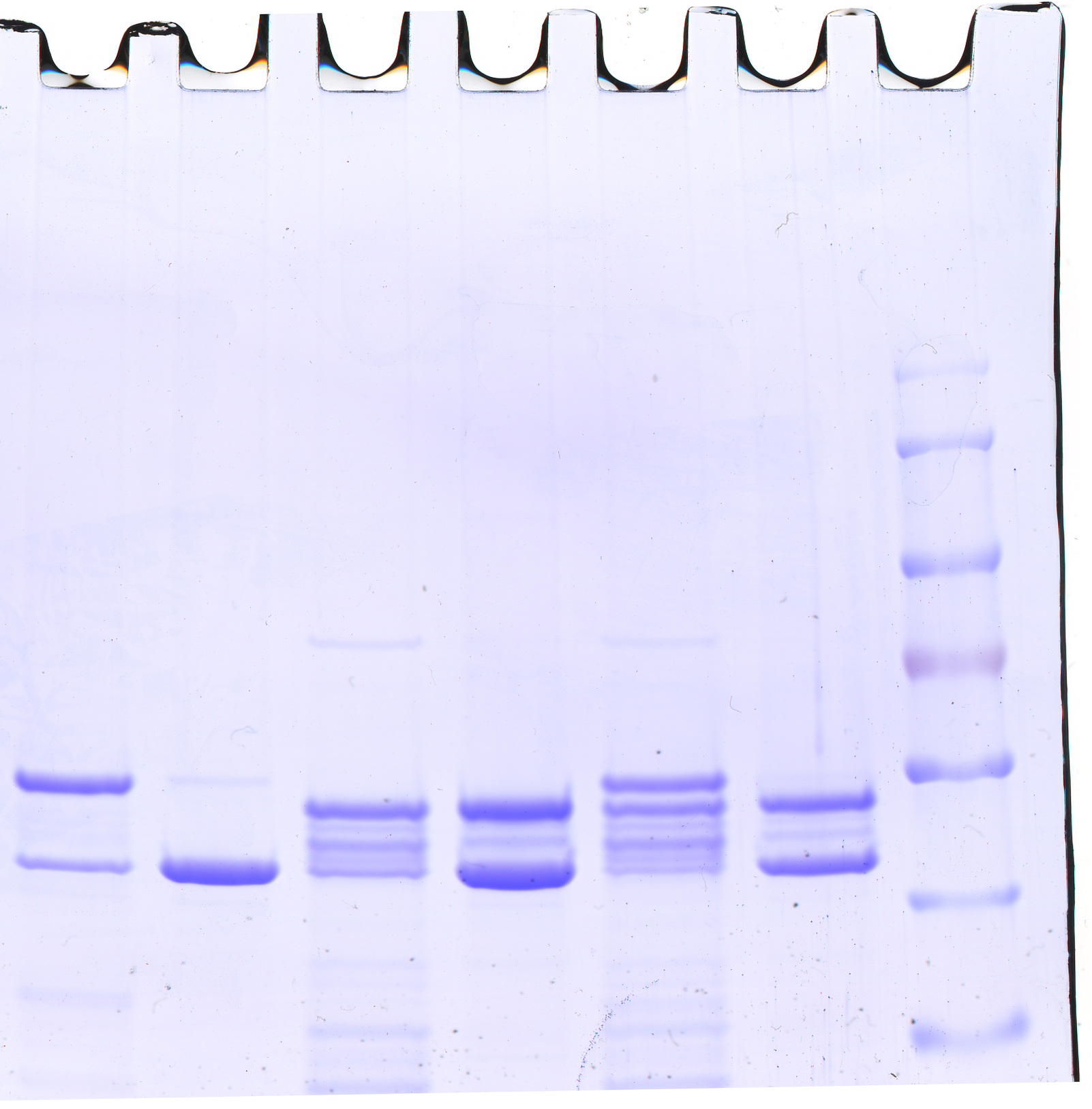

Supplement: Figure 5—source data 1. [file elife-93662-fig5-data1.zip › Figure 5-Source data 1/Original files for Figure 5A/MlphABD cosedimentation with Actin with or without Myo5a-MTD .tif]

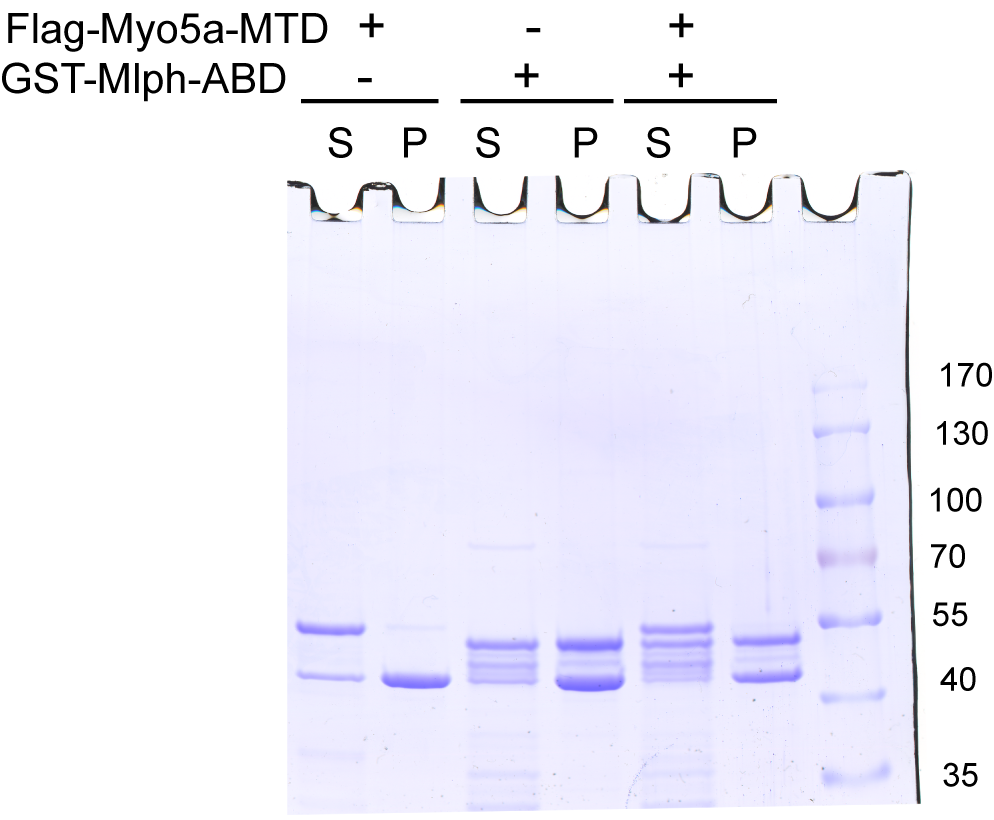

Supplement: Figure 5—source data 1. [file elife-93662-fig5-data1.zip › Figure 5-Source data 1/Uncropped gels for Figure 5A/MlphABD cosedimentation with Actin with or without Myo5a-MTD .tif]

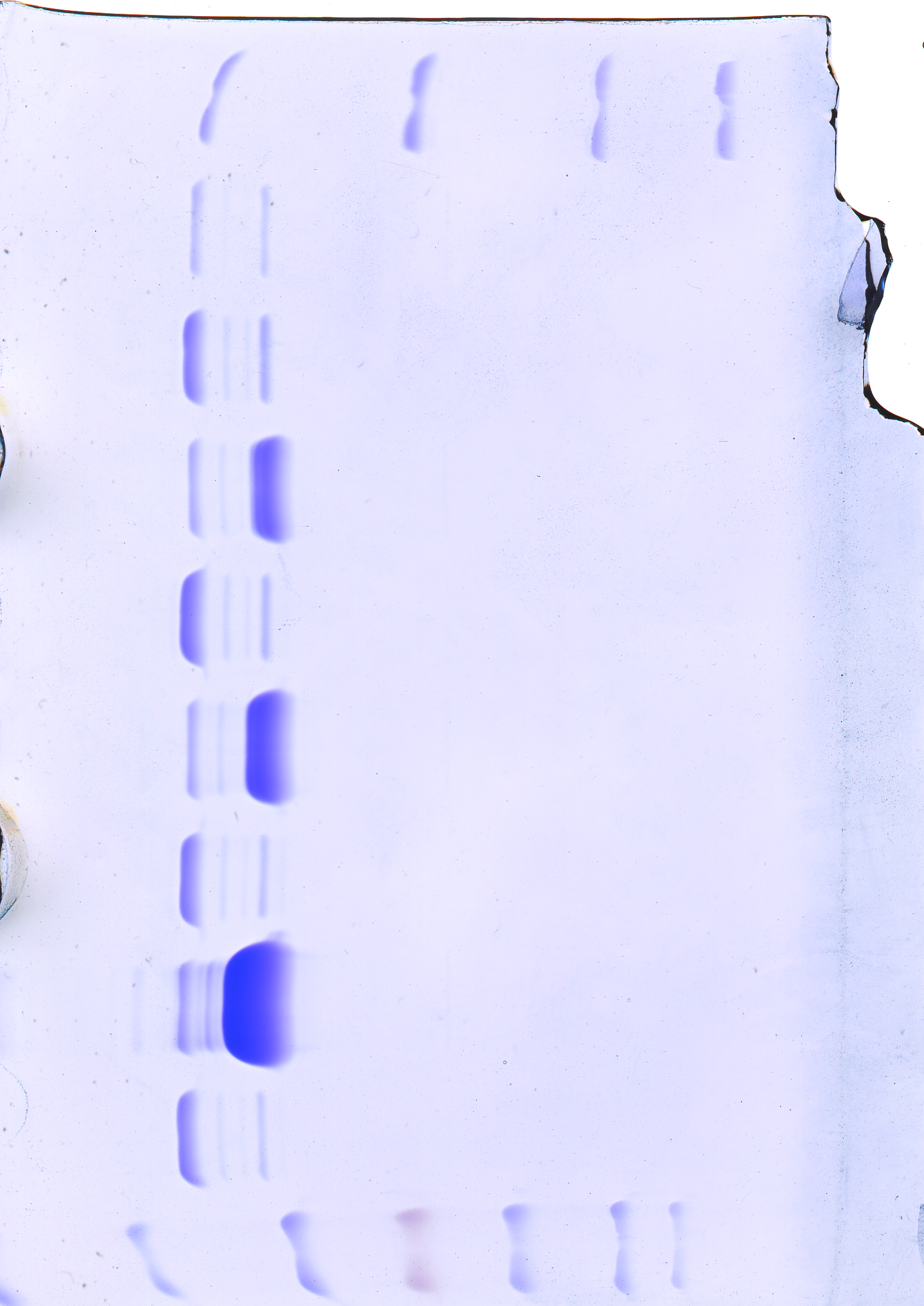

Supplement: Figure 5—source data 2. [file elife-93662-fig5-data2.zip › Figure 5-Source data 2/Original files for Figure 5B/MlphABD cosedimentation with different Myo5a-MTD conc.tif]

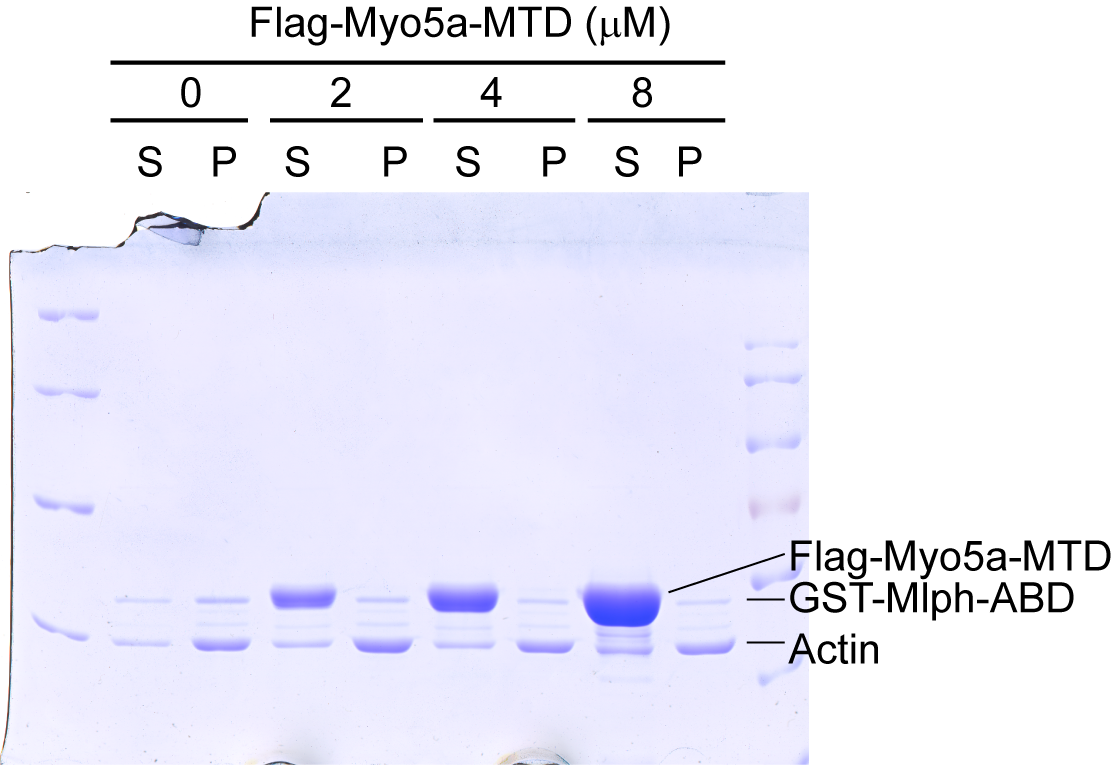

Supplement: Figure 5—source data 2. [file elife-93662-fig5-data2.zip › Figure 5-Source data 2/Uncropped gels for Figure 5B/MlphABD cosedimentation with different Myo5a-MTD conc.tif]

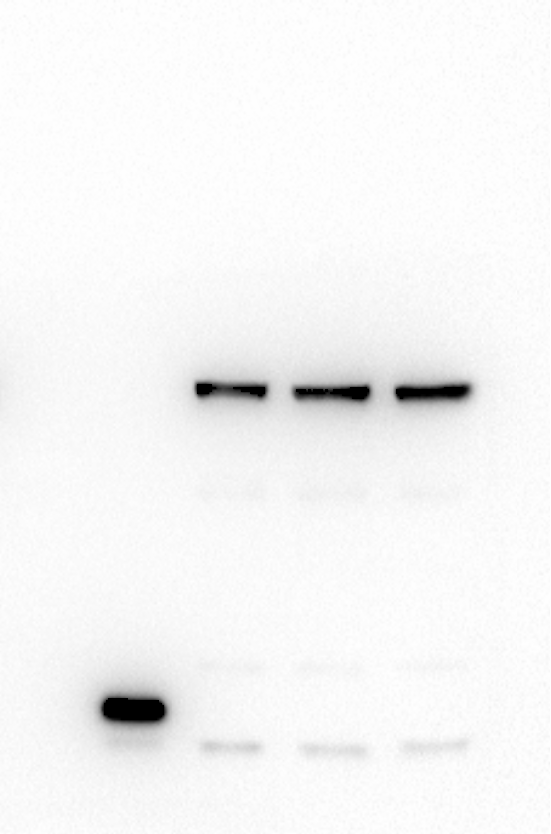

Supplement: Figure 6—source data 2. [file elife-93662-fig6-data2.zip › Figure 6-Source data 2/Original files for Figure 6C/EGFP.tif]

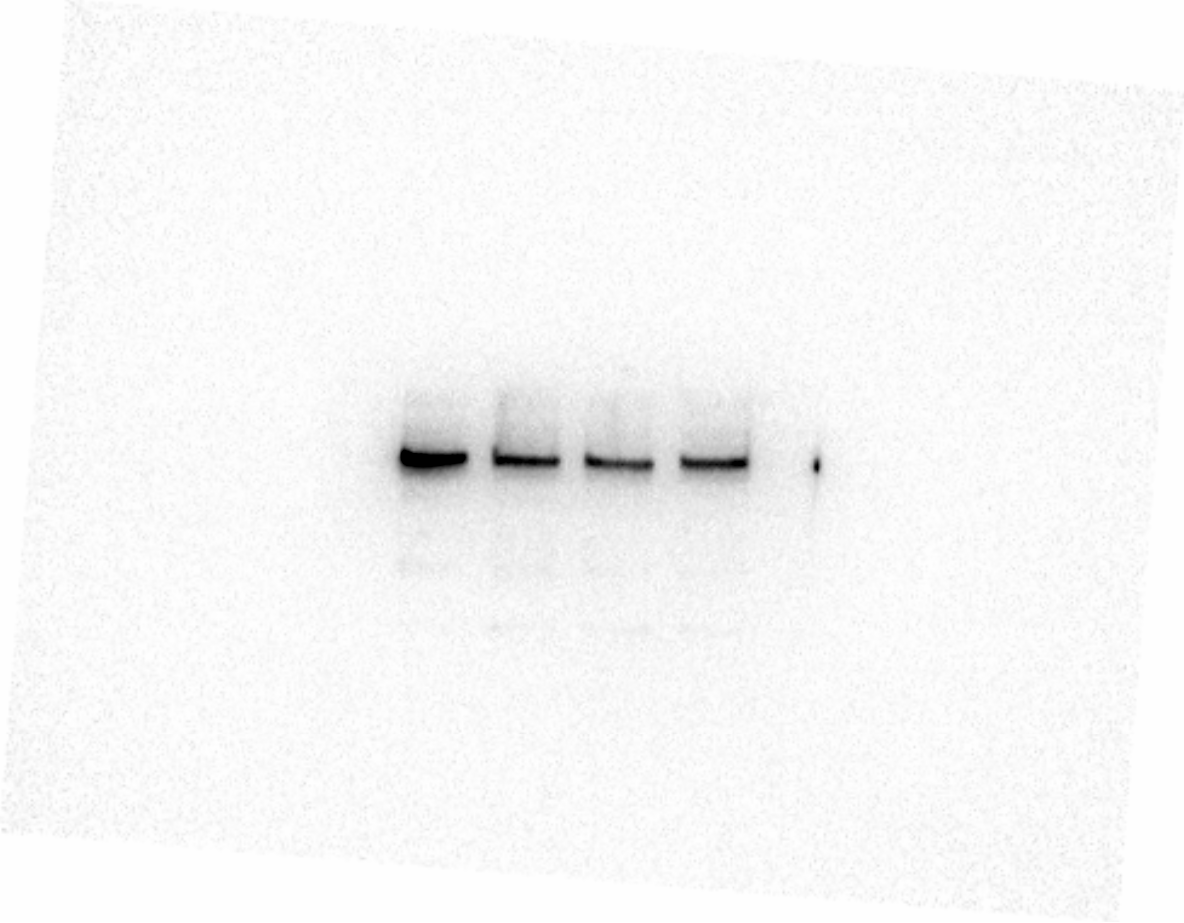

Supplement: Figure 6—source data 2. [file elife-93662-fig6-data2.zip › Figure 6-Source data 2/Original files for Figure 6C/β-tubulin.tif]

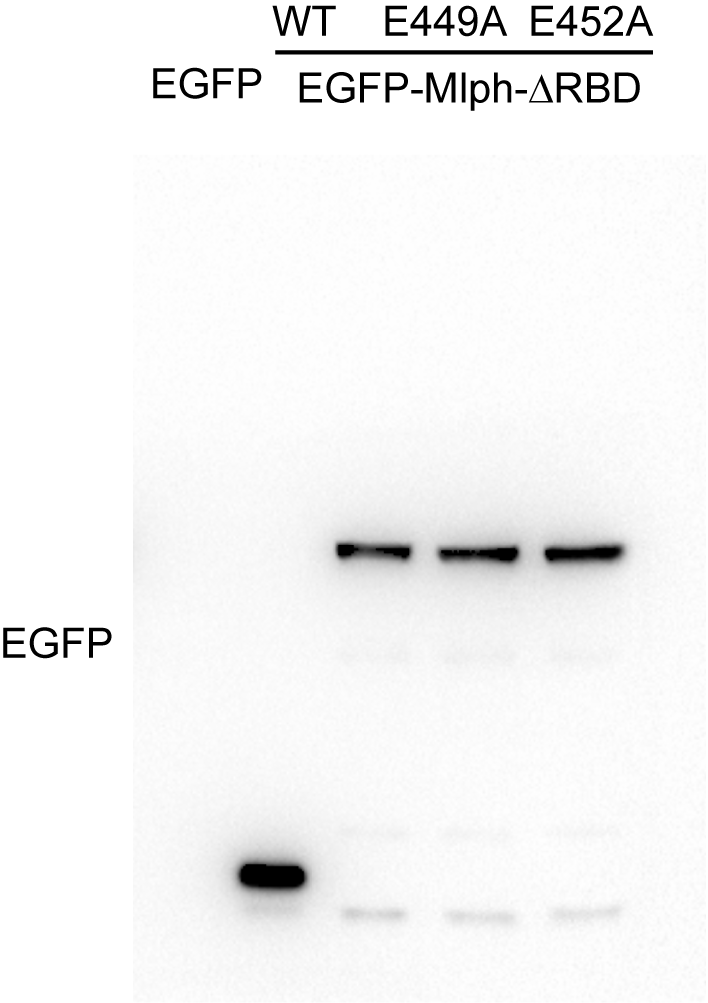

Supplement: Figure 6—source data 2. [file elife-93662-fig6-data2.zip › Figure 6-Source data 2/Uncropped blots for Figure 6C/EGFP.tif]

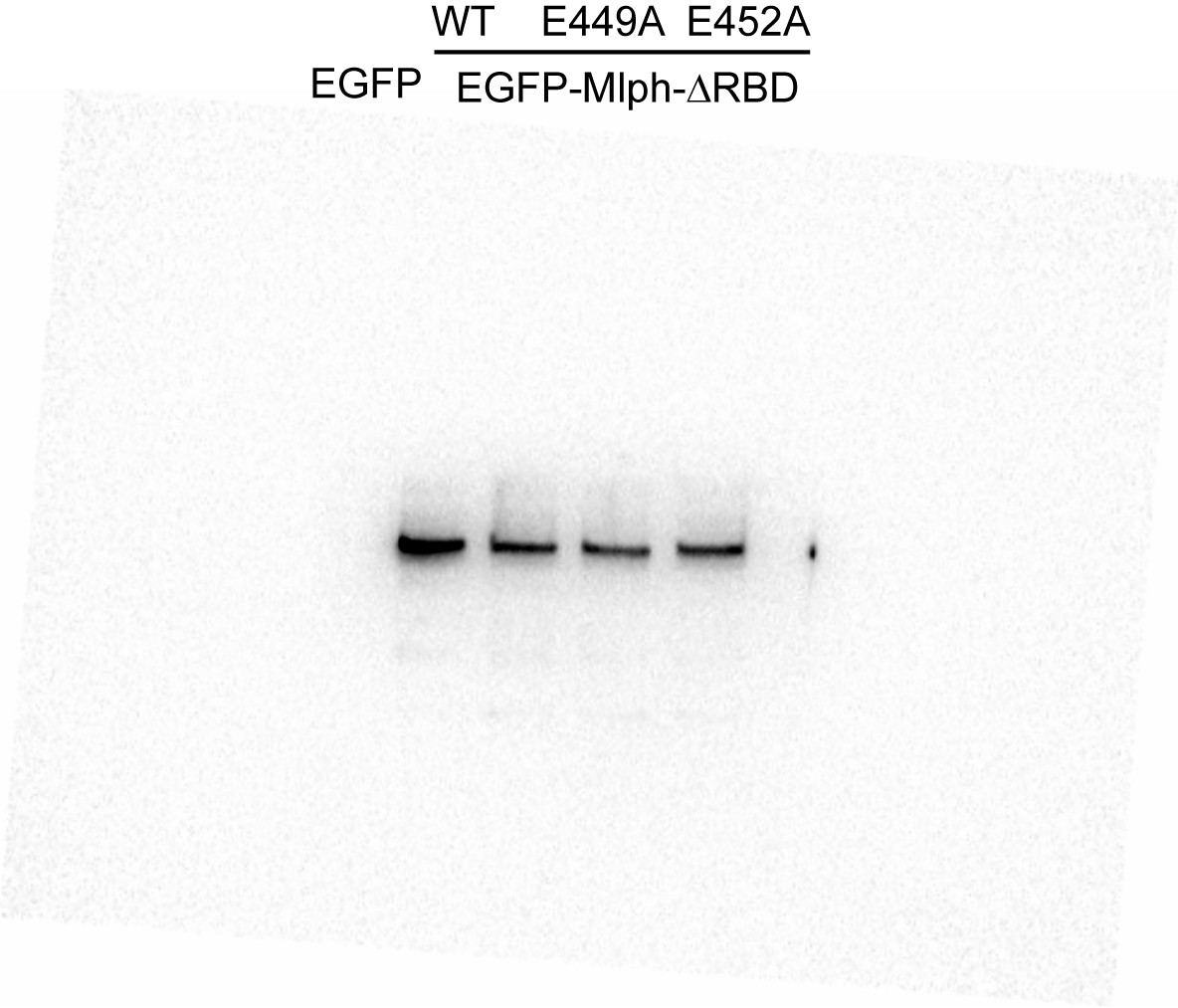

Supplement: Figure 6—source data 2. [file elife-93662-fig6-data2.zip › Figure 6-Source data 2/Uncropped blots for Figure 6C/β-tubulin.tif]

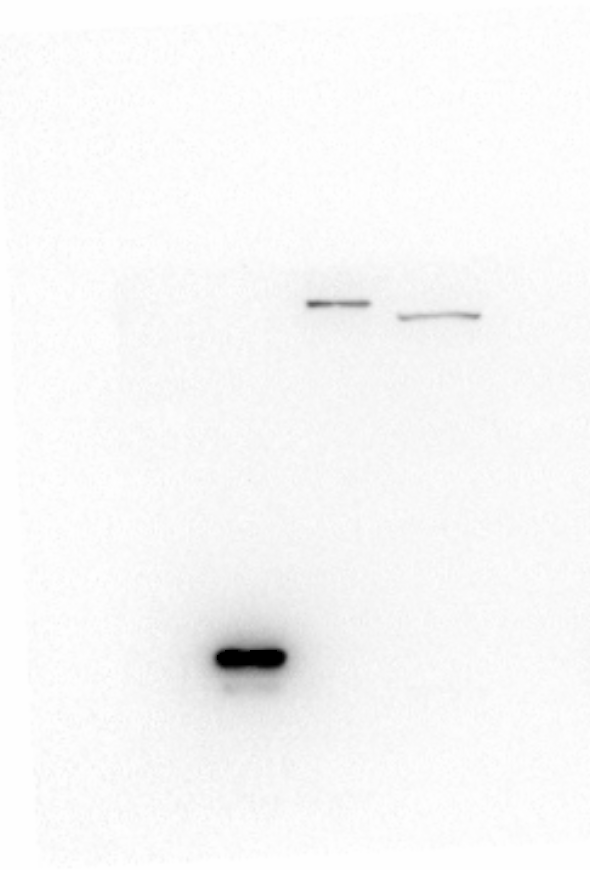

Supplement: Figure 7—source data 2. [file elife-93662-fig7-data2.zip › Figure 7-Source data 2/Original files for Figure 7D/EGFP.tif]

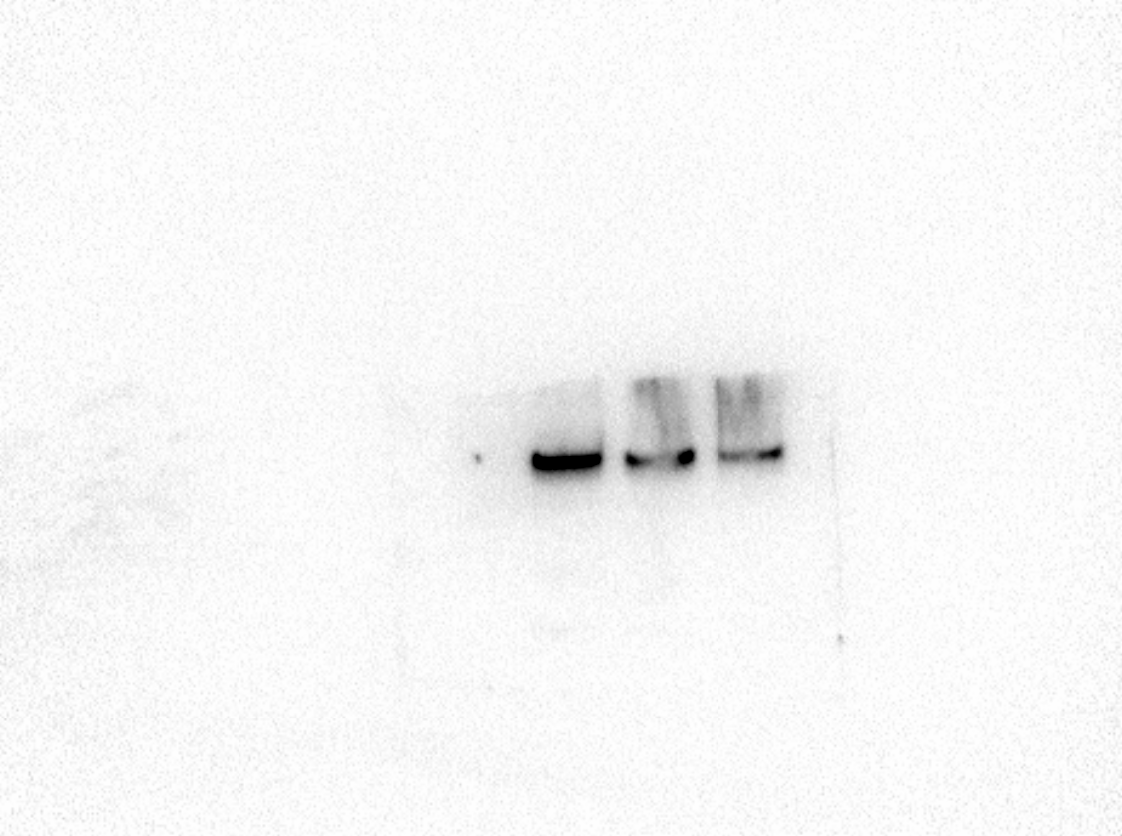

Supplement: Figure 7—source data 2. [file elife-93662-fig7-data2.zip › Figure 7-Source data 2/Original files for Figure 7D/β-tubulin.tif]

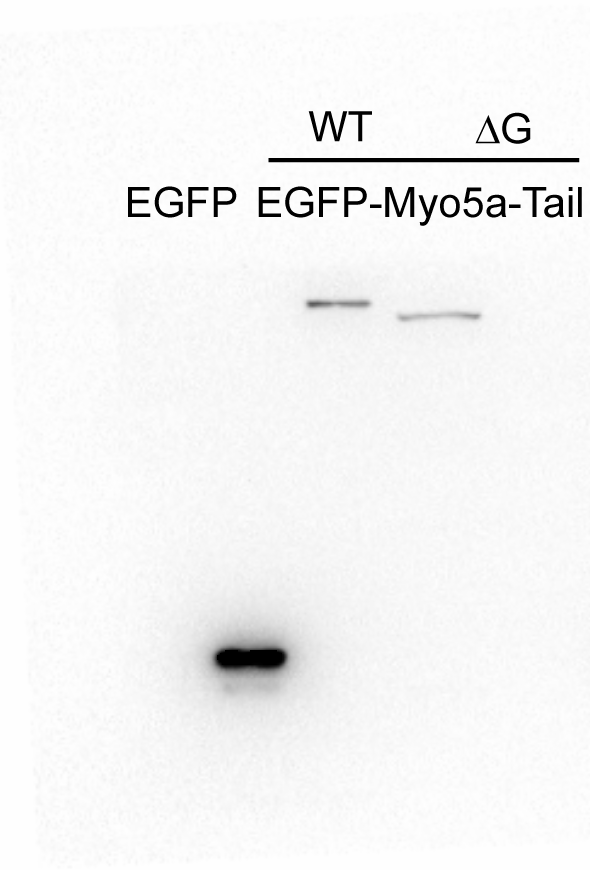

Supplement: Figure 7—source data 2. [file elife-93662-fig7-data2.zip › Figure 7-Source data 2/Uncropped blots for Figure 7D/EGFP.tif]

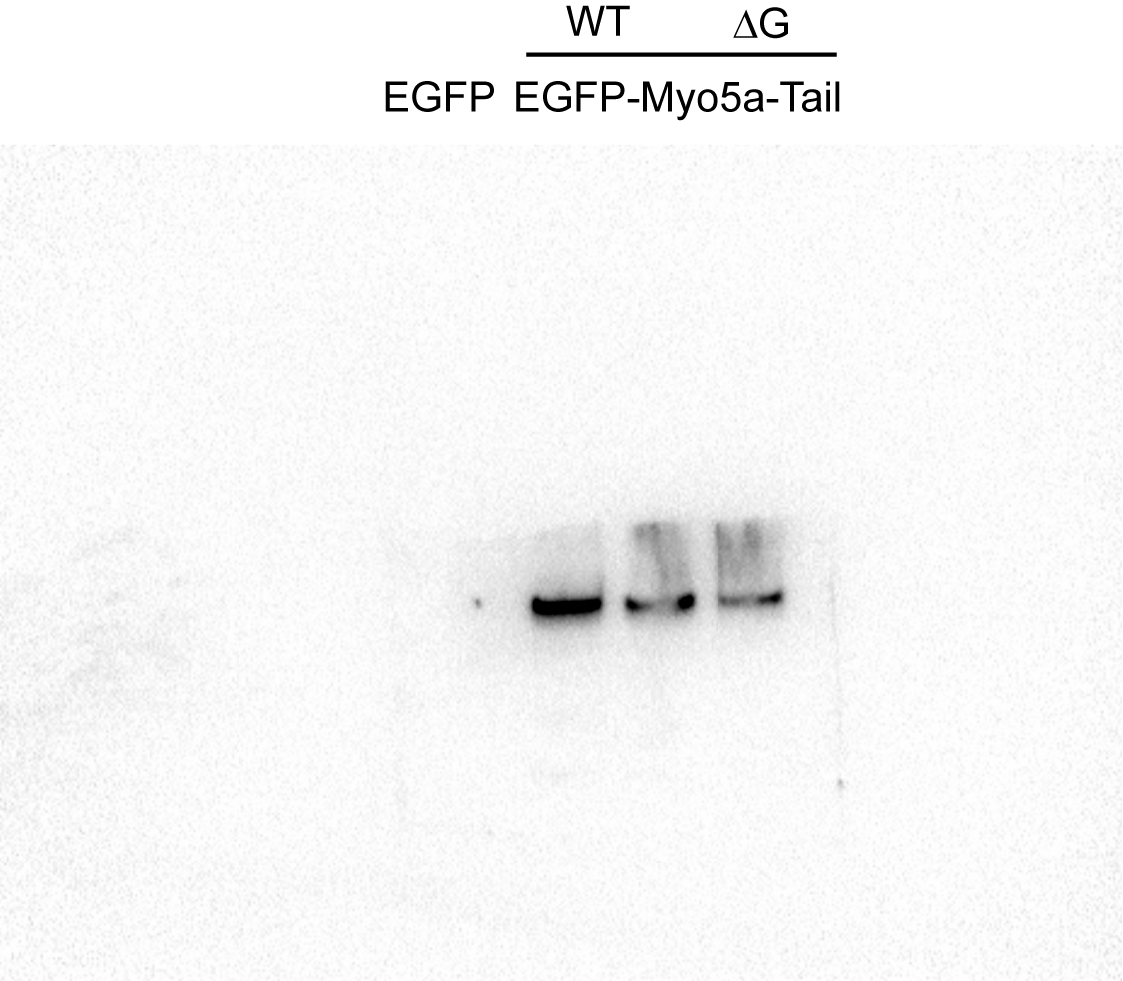

Supplement: Figure 7—source data 2. [file elife-93662-fig7-data2.zip › Figure 7-Source data 2/Uncropped blots for Figure 7D/β-tubulin.tif]
